# Spatiotemporally controlled, aptamers-mediated growth factor release locally manipulates microvasculature formation within engineered tissues

**DOI:** 10.1016/j.bioactmat.2021.10.024

**Published:** 2021-10-23

**Authors:** Deepti Rana, Ajoy Kandar, Nasim Salehi-Nik, Ilyas Inci, Bart Koopman, Jeroen Rouwkema

**Affiliations:** a Department of Biomechanical Engineering, Technical Medical Centre, Faculty of Engineering Technology, University of Twente, 7500, AE, Enschede, the Netherlands; b Izmir Democracy University, Vocational School of Health Services, Department of Dentistry Services, Dental Prosthetics Technology, Izmir, 35140, Turkey

**Keywords:** Aptamers, Vascular endothelial growth factor, Spatiotemporal release, Co-culture, Vascularization, Biomaterials, Tissue engineering

## Abstract

Spatiotemporally controlled growth factor (GF) delivery is crucial for achieving functional vasculature within engineered tissues. However, conventional GF delivery systems show inability to recapitulate the dynamic and heterogeneous nature of developing tissue's biochemical microenvironment. Herein, an aptamer-based programmable GF delivery platform is described that harnesses dynamic affinity interactions for facilitating spatiotemporal control over vascular endothelial GF (VEGF_165_) bioavailability within gelatin methacryloyl matrices. The platform showcases localized VEGF_165_ sequestration from the culture medium (offering spatial-control) and leverages aptamer-complementary sequence (CS) hybridization for triggering VEGF_165_ release (offering temporal-control), without non-specific leakage. Furthermore, extensive 3D co-culture studies (human umbilical vein-derived endothelial cells & mesenchymal stromal cells), in bi-phasic hydrogel systems revealed its fundamentally novel capability to selectively guide cell responses and manipulate lumen-like microvascular networks via spatiotemporally controlling VEGF_165_ bioavailability within 3D microenvironment. This platform utilizes CS as an external biochemical trigger for guiding vascular morphogenesis which is suitable for creating dynamically controlled engineered tissues.

## Highlights

• Spatiotemporally controlled VEGF_165_ bioavailability within bi-phasic acrydite-modified aptamer-functionalized hydrogels.
• Aptamer-bound-VEGF_165_ within bi-phasic hydrogels revealed their ability to selectively guide cell responses.
• This unique behavior could be harnessed for regulating vascular morphogenesis within engineered tissues in 4D.
• With complementary sequences as an external trigger, dynamically controlled mature microvascular networks were observed.

## Graphical abstract

**Figure:**
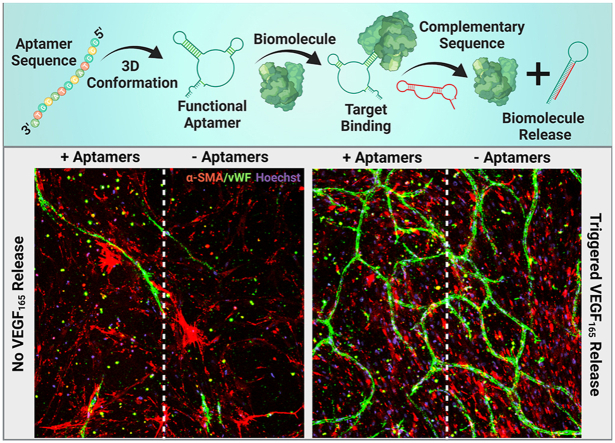

## 1 Introduction

A long-sought goal of tissue engineering is to successfully bioengineer artificial tissues that could repair or regenerate damaged tissues after implantation in patients. However, establishing hierarchically organized, perfusable and mature vascular networks within engineered tissues is fundamental for their survival post-implantation. [1]. In native tissues, various processes are synergistically responsible for achieving vascularization, including vasculogenesis (*de novo* vessel formation), angiogenic sprouting, intussusception, vascular remodeling and maturation. Similar to other tissue formation processes, vascularization is also spatiotemporally controlled via various interactions of the cells with the extracellular matrix (ECM) which undergoes constant biochemical modifications. [2]. The complex, dynamic and interdependent nature of these interactions in native tissues provides great challenges in tissue engineering, but also highlights the need to design dynamic biomaterials that could spatiotemporally control various biochemical cues for better mimicking the *in vivo* microenvironment.

Spatiotemporally controlled biochemical cues (growth factor, GF) within biomaterials provide unique opportunities for governing defined cell behaviors such as adhesion, proliferation, migration, and differentiation. [[3], [4], [5], [6], [7], [8], [9], [10]]. Recent research has demonstrated a variety of bioinspired designs that manifest the ability to temporally control GF delivery by being transiently activated with external stimuli such as pH, enzymes, light, abiotic avidin affinity, cell-selective traction forces, or a logic-based integrated combination of multiple stimuli. [[11], [12], [13], [14], [15], [16], [17], [18], [19], [20], [21]]. Although these approaches have their own merits and show efficient functionality *in vitro*, they have limited *in vivo* applicability. For instance, light-dependent approaches exhibit significant light scattering and lower absorption at higher tissue depths. [22]. Indeed, diffusion-dependent approaches such as pH leverages on temporal control within deep tissue, but shows suboptimal spatial selectivity. Moreover, synthetic approaches like abiotic-affinity designs, logic-based multiple stimulus combination, cell-selective traction force and matrix metalloproteinase based-triggers overcome the issues of spatial selectivity, but often impose challenges including complex system designs, low cost-effectiveness, or rely on protein modifications of the GF. Approaches enabling spatial control over GF delivery via covalent immobilization, covalent tethering with stimuli-sensitive linkers (such as pH, enzymes, light), or protein modification, also have their own limitations. For example, covalent immobilization inhibits GF internalization that could inadvertently interfere with the biological processes. [23]. Besides, it is challenging to maintain conformations and/or biological activity after conjugation (or during the process) within polymer matrices. [23].

To address the aforementioned limitations and enable high specificity along with controlled therapeutic release, we sought to develop a versatile aptamer-based GF delivery platform that (i) is highly selective for rapid GF sequestration, (ii) enables on-demand triggered GF release via complementary sequence (CS) hybridization, (iii) decouples GF loading and hydrogel formation (avoiding harsh conditions or GF modifications), thus promising maximum bioactivity, (iv) is suitable for exploring 3D cell encapsulation (specifically, for tissue engineering applications), and (v) is capable of guiding cell behaviors such as survival, attachment, migration and proliferation, by spatiotemporally controlling GF bioavailability in 3D. In our rational approach, we set out to harness the unique capabilities of aptamers which are small, single-stranded oligonucleotides selected from synthetic RNA/DNA libraries. [24]. Depending on sequence, oligonucleotides form unique 3D conformations that enable selective binding to target biomolecules with high affinity and specificity, thus minimizing risk of non-specific release. [24]. While oligonucleotides are naturally susceptible to DNases degradation *in vivo*, approaches such as aptamer PEGylation and RNA-modified oligonucleotides have significantly reduced DNase-mediated degradation *in vivo*. [25]. Consequently, the clinically approved vascular endothelial growth factor (VEGF)-binding RNA-based aptamer, Pegaptanib sodium, which is used for macular degeneration treatment (Macugen, Eyetech/Pfizer), utilizes both PEGylation and oligonucleotide modification to achieve an *in vivo* half-life of 10 (±4)days. [25]. Moreover, aptamers can be designed for higher affinity to CS than the target GF resulting in aptamer-CS binding via intermolecular hybridization, thus enabling on-demand GF release using CS as an external trigger. [24]. It has been shown that biomaterials (or polymeric particles) functionalized with aptamers specific for different GFs (for example, VEGF and platelet-derived growth factor BB (PDGF-BB) or basic fibroblast growth factor) [[26], [27], [28]], [[26], [27], [28]] [[26], [27], [28]] can sustain sequential release of multiple GFs via aptamer-CS hybridization.

Even though aptamer based biomaterials have been reported in literature, so far their bioactivity has been mainly studied through indirect approaches. Prior research with endothelial cells or smooth muscle cells cultured using medium conditioned with GFs (VEGF and/or PDGF-BB) released from aptamer-functionalized hydrogels, reported enhanced survival of cells (>60%) compared to controls. [26]. Moreover, *in vivo* studies using their injectable formulations for dual GF delivery showed higher GF retention, no cytotoxicity and increased blood vessel formation. However, the study involved aptamer-bound-GF delivery coupled with polymer degradation and ECM remodeling *in vivo*. [26]. Although, previous literature demonstrates the valuable potential of aptamer-functionalized biomaterials, they did not evaluated the direct bioactivity of aptamer-bound-GF within the biomaterial and its effect on cellular responses. Therefore, in order to explore the complete potential of aptamer-functionalized biomaterials, specifically for regenerating tissues, it is important to systematically study their interaction with cells within 3D microenvironment.

## 2 Materials and methods

### 2.1 Materials

Type A 300 bloom porcine skin gelatin (G1890-500G, Sigma Aldrich), Methacrylic anhydride (MA, 276685-500 ML, Sigma Aldrich), Dulbecco's phosphate buffered saline (DPBS, D8537-500 ML, Sigma Aldrich), Fisherbrand™ regenerated cellulose dialysis tubing (12–14 kDa, 21-152-14, Fisher Scientific), bovine serum albumin (BSA, A9418, Sigma Aldrich), deuterium oxide (151890, Sigma-Aldrich), 2-hydroxy-4’-(2-hydroxyethoxy)-2-methylpropiophenone (Irgacure 2959, 410896, Sigma Aldrich), VEGF specific control aptamer (47-nt, DNA, IDT), 5′ acrydite modified aptamer (47-nt, DNA, IDT), complementary sequence (Comp. Seq., 46-nt, DNA, IDT), 5′Alexa Fluor 488 modified complementary sequence (Fluoro-Comp. Seq., 46-nt, DNA, IDT), nuclease free water (11-04-02-01, IDT), Fluoro-Max dyed blue aqueous fluorescent particles (2 μm diameter) (B0200, Thermo Scientific), human VEGF ELISA kit (RAB0507-1 KT, Sigma Aldrich), glutaraldehyde solution (340855, Sigma Aldrich), human umbilical vein endothelial cells (HUVECs, C2519A, Lonza), human mesenchymal stromal cells (MSC, PT-2501, Lonza), α –MEM medium (+nucleosides, 22571-020, Gibco), fetal bovine serum (FBS, F7524, Sigma), GlutaMax™ supplement (35050061, Gibco), penicillin-streptomycin (pen/strep, 15140-122, Gibco), l-abscorbic acid (A8960, Sigma Aldrich), trypsin-EDTA 0.25% (+phenol red, 25200072, Gibco), vascular endothelial growth factor 165 human (VEGF, H9166, Sigma Aldrich), Gibco™ FGF-Basic AA 1–155 recombinant human protein (bFGF, PHG0264, Fisher Scientific), endothelial cell basal medium 2 (EGM 2, C-22211, PromoCell), endothelial cell growth medium 2 supplement pack (EGM 2, C-39211, PromoCell), PDMS silicone elastomer (2401673921, Sylgard), live/dead cell double staining kit (04511, Sigma Aldrich), formaldehyde solution (F8775-25 ML, Sigma Aldrich), Triton™ X-100 (T8787, Sigma Aldrich), Phalloidin-Atto 488 (49409, Sigma Aldrich), Invitrogen™ Alexa Fluor™ 647 Phalloidin (A22287, Fisher Scientific), Invitrogen™ hoechst 33342 (H1399, Fisher Scientific), monoclonal anti-actin, α-smooth muscle antibody (mouse, A2547, Sigma Aldrich), anti-von Willebrand factor antibody (rabbit, F3520, Sigma Aldrich), human VE-Cadherin antibody (mouse, MAB9381, R&D systems), IgG (H + L) cross-adsorbed goat anti-rabbit, Alexa Fluor® 488, Invitrogen™ (A11008, Fisher Scientific), IgG (H + L) cross-adsorbed goat anti-mouse, Alexa Fluor® 488, Invitrogen™ (A11001, Fisher Scientific), IgG (H + L) highly cross-adsorbed goat anti-mouse, Alexa Fluor® 594, Invitrogen™ (A11032, Fisher Scientific), Alexa Fluor® 647 Anti-VEGFA antibody [EP1176Y] (ab206887, abcam), Corning® 96 well black polystyrene microplate (CLS3603, Sigma), Corning® Costar® ultra-low attachment well plates were used for all cell culture experiments (CLS3473, CLS3474, Sigma).

### 2.2 Prediction of secondary structures

The secondary structures of DNA based aptamers and their 3D conformations were generated using NUPACK. The presented structure with the lowest free energy at 37 °C was presumed to be the dominant structure (Fig. S1).

### 2.3 Synthesis and characterization of gelatin methacryloyl

Gelatin methacryloyl (GelMA) was synthesized as described previously elsewhere with medium degree of methacryloyl substitution (∼60%). [29]. Briefly, gelatin was dissolved at 10% (w/v) into DPBS at 60 °C and stirred until fully dissolved. Subsequently, 1.25% (v/v) of MA was added at a rate of 0.5 ml/min to the gelatin solution at 50 °C for 1hr with continuous stirring. Afterwards, the reaction was stop with additional warm (40 °C) DPBS leading to 5x dilution. The solution was then dialyzed against distilled water using 12–14 kDa cutoff dialysis tubing at 40 °C for 1 week to remove residual salts and methacrylic acid. The solution was freeze-dried for 1 week and stored at −20 °C until further use. The degree of functionalization was quantified by H^1^ NMR. [29]. The H^1^ NMR spectra were collected at 35 °C in deuterium oxide at a frequency of 400 MHz using NMR spectrometer. The obtained spectral data was analyzed using MestReNova software (Fig. S2).

### 2.4 Synthesis of aptamer-functionalized hydrogels

The freeze-dried GelMA macromer was mixed with Irgacure 2959 as a photoinitiator in DPBS at 60 °C until fully dissolved. Afterwards, VEGF_165_ specific aptamers (control aptamer or acrydite aptamer, see Table S1) reconstituted in nuclease free water, was added into this solution with 5% (w/v) GelMA, 0.5% (w/v) Irgacure 2959 and 2.5 nmoles of aptamers in every 50 μl of pre-polymer solution. This pre-polymer solution was then used to prepare hydrogel samples for different experiments. Additionally, plain GelMA hydrogels were synthesized using same pre-polymer solution but without aptamer. For photo-crosslinking, requisite amount of pre-polymer solution was added into a PDMS mold, covered with a coverslip and exposed to 0.23 mW/cm^2^ intensity UV light (360–480 nm) (UV-KUB 2, Kloe, France) for 2 min. Samples were detached from the coverslips, transferred to their respective well plates using sterilized spatulas and used for different experiments.

### 2.5 Evaluation of aptamer retention within the hydrogels

To evaluate aptamer incorporation and their retention within the hydrogels, the aptamer-functionalized (acrydite-aptamer or control-aptamer) hydrogels and GelMA hydrogels were prepared by adding 25 μl of pre-polymer solution into PDMS mold (2 mm thickness and 4 mm diameter) following 2mins of UV crosslinking. Post photo-crosslinking, the hydrogels were transferred into clear-bottom black polystyrene 96-well plate with one hydrogel per well and were incubated with 2.5nmoles of Alexa Fluor-488 labelled complementary sequence (Fluoro-CS) that binds to the VEGF_165_ specific aptamer for 24hrs in 100 μl DPBS. A mole ratio of 1:1 for aptamer to CS was maintained. After 24hrs, the supernatant was discarded and the hydrogels were washed once with DPBS to remove unbound CS. To this end, 100 μl of fresh DPBS was added into the hydrogel and imaged using fluorescence microscope (EVOS M7000, Thermo Fisher Scientific) as day1 for the experiment. The experiment was carried out for 10days where after every 24hrs, the hydrogel's supernatant was replaced with fresh 100 μl DPBS. The hydrogels were imaged at 60% intensity & 120 ms exposure time; 2x objective. The experiment was performed with three experimental replicates for each group. To quantify the fluorescence intensity, ImageJ (NIH, USA) software was used.

### 2.6 Analysis of VEGF_165_ sequestration and its triggered release

To conduct VEGF_165_ sequestration and triggered release experiments, the aptamer-functionalized hydrogels (2.5nmoles of acrydite-aptamer or control-aptamer) were prepared by adding 50 μl of pre-polymer solution into a PDMS mold (2 mm thickness & 6 mm diameter) following 2mins of UV crosslinking. Additionally, plain GelMA hydrogels without aptamers were prepared. Once crosslinked, the hydrogels were transferred to ultra-low attachment 24-well plates. For VEGF_165_ loading, the hydrogels were incubated with 1 ml of releasing medium (0.1% BSA in DPBS) having VEGF_165_ (10 ng) for 1hr at 37 °C. The mole ratio of aptamer to VEGF_165_ was ∼10000:1. After 1hr incubation, the supernatant was removed and the hydrogels were washed once with releasing medium. Afterwards, the VEGF_165_ loaded hydrogels were incubated in 1 ml releasing medium. After every 24hrs, the supernatant was removed and fresh 1 ml releasing medium was added until day10. The VEGF retention was determined by subtracting the amount of free VEGF_165_ in loading solution after 1hr incubation from the initial loaded VEGF_165_ amount. The VEGF_165_ loaded aptamer-functionalized hydrogels were also examined for their on-demand triggered release behavior. For this purpose, on day4, 2.5nmoles of CS (complementary to VEGF_165_ specific aptamer) was added to releasing medium making a final volume of 1 ml into “acrydite aptamer + CS@D4 & D9” & “Control aptamer + CS@D4 & D9” samples for 24hrs. However, in all other hydrogels only releasing medium (no CS) was added on day4. Furthermore, on day9, 2.5nmoles CS was added making final volume of 1 ml releasing medium to all aptamer-functionalized hydrogel samples. The triggered VEGF_165_ release was determined by the VEGF_165_ amount released on day5 and day10 (within 24hrs of CS addition). All of the supernatants, including loading and washing solutions were stored at −20 °C until further analysis. In order to evaluate the efficacy of ELISA assay in identifying VEGF_165_ molecules bound to control-aptamer in supernatant solution, separate experiment was designed. For this purpose, 2.5nmoles control-aptamer and VEGF_165_ (10 ng) was directly added into 1 ml releasing medium and allowed to incubate for 24hrs at 37 °C. As a control, only VEGF_165_ was added into 1 ml releasing medium, without control-aptamers. The ultra-low attachment 24-well plate was used for this experiment to avoid protein adsorption by the well plate. After 24hrs, the solution was collected and stored at −20 °C. The amount of VEGF_165_ present in supernatants was measured by VEGF_165_ ELISA kit as per manufacturer's instructions. The absorbance for the samples were measured using microplate reader (Infinite® 200PRO, Tecan) at 405 nm. Prior to analysis, supernatants were diluted with the sample diluent to ensure the VEGF_165_ concentrations within the detectable range of the assay. The absorbance was referenced by subtracting it from the absorbance of zero VEGF_165_ concentration. The ELISA data was analyzed using GraphPad Prism software. The experiment was performed with three experimental replicates.

### 2.7 Scanning electron microscopy

For scanning electron microscopy (SEM) analysis, aptamer-functionalized hydrogels with different (acrydite- or control-) aptamer amounts (0.25nmoles, 2.5nmoles & 25nmoles) were prepared as previously discussed. The GelMA hydrogel without any aptamer was prepared as control. For preparing the hydrogel, 500 μl of pre-polymer solution was added into 24-well plate followed by 2mins of UV crosslinking. The hydrogel discs of about ∼4 mm thickness and 15 mm diameter were obtained. The hydrogels were washed with DPBS followed by fixing in 2.5% glutaraldehyde for 24hr at 4 °C. Afterwards, the hydrogels were flash-freezed in liquid nitrogen and freeze-dried for 4days. The freeze-dried hydrogel discs were broken in liquid nitrogen to observe the cross-section, gold-sputtered (Sputter Coater 108 Auto, Cressington Scientific Instruments) and imaged using SEM (JSM-IT100, JEOL). Considering the large hydrogel disc size, three different regions from the same sample were imaged for pore size measurement within each group using ImageJ software.

### 2.8 Rheological analysis

The viscoelastic properties analysis of the aptamer-functionalized hydrogels with different (acrydite- or control-) aptamer amounts (0.25nmoles, 2.5nmoles & 25nmoles) were performed using parallel plate geometry (PP8, 8 mm) on a stress-controlled rheometer (Physica MCR301, Anton Paar). The parallel plate and bottom rheometer plate were treated with suitable sandpaper to prevent slipping. The GelMA hydrogel was prepared as control. Post UV crosslinking, hydrogel samples (8 mm diameter and 1 mm thickness) were placed onto the rheometer plate at ambient room temperature (20 °C) and the parallel plate was lowered to the desired gap height of 1 mm. A customized 3D-printed solvent trap was used to prevent evaporation during the rheological measurement. The amplitude sweep measurement at a constant 1 rad/s angular frequency (ω) was carried out over the range of 0.1% till 100% strain (γ) to examine viscoelastic properties of hydrogels. The storage modulus (G’) and loss modulus (G”) were determined as the strain (%) changes during oscillatory shear. The elastic modulus of hydrogel was determined from the linear region of amplitude sweep data.

### 2.9 Cell culture

The human umbilical vein-derived endothelial cells (HUVECs, Lonza) and human mesenchymal stromal cells (MSCs, Lonza) were cultured as per the standard protocol. In brief, HUVECs were cultured in EGM-2 medium (EGM-2 Basal medium + EGM-2 Supplements) with 1% (v/v) Pen/Strep. However, for MSCs, α-MEM medium supplemented with 10% (v/v) FBS, 2 mM l-glutamine, 0.2 mM ascorbic acid, 1% (v/v) Pen/Strep and 1 ng/ml bFGF was used. Both cell types were cultured in a humidified atmosphere with 5% CO_2_ at 37 °C and passaged till 80% confluence.

### 2.10 3D Co-culture in aptamer-functionalized hydrogels

For co-culture experiments, 1:1 ratio of HUVECs to MSCs with a total seeding density of 2.5 × 10^6^ cells ml^−1^ was used throughout this study. Similarly, MSCs medium (α-MEM, 10% FBS, 2 mM l-glutamine, 0.2 mM abscorbic acid & 1%Pen/Strep) and HUVECs medium (EGM-2 Basal medium+1%Pen/Strep + all EGM-2 supplements except for VEGF_165_ (C-30260, PromoCell)) were used in 1:1 ratio as co-culture medium without VEGF_165_ supplement. In all experiments, both cell types between passage 3 and 5 were used. For 3D co-culture experiments, HUVECs (P3) and MSCs (P4) were trypsinized, counted (using trypan blue) and were re-suspended in 1:1 ratio with total seeding density of 2.5 × 10^6^ cells ml^−1^. This cell suspension was further centrifuged at 300g for 3mins at 4 °C to get the cell pellet. The supernatant was removed and pre-polymer solution (5%GelMA+0.5% Irgacure+2.5nmoles acrydite- or control-aptamer in DPBS) was directly added and mixed gently by pipetting. 50 μl from this pre-polymer solution with cells was dispensed into ultra-low attachment 96-well plate, followed by 2mins of UV crosslinking. The experiment was performed under aseptic conditions. Afterwards, 200 μl of co-culture medium along with VEGF_165_ (10 ng) was added onto the hydrogels and were kept inside humidified incubator at 37 °C with 5% CO_2_ for 1hr. After 1hr of VEGF_165_ loading, supernatant was removed, hydrogels were washed twice with DPBS and then 200 μl of co-culture medium was added. The medium was changed after every 24hrs throughout the study duration. As a control, GelMA hydrogel with cells encapsulated in it were also prepared similarly without the aptamer. To observe the effect of triggered release of VEGF_165_ on the cells, 2.5nmoles of CS (with aptamer to CS ratio of 1:1) was added onto the 200 μl of co-culture medium on day4. These hydrogels were analyzed for cell viability on day1 and day5 by using Live/Dead cell viability kit as per manufacturer's protocol. Briefly, the culture medium of the hydrogel was removed and 100 μl of staining solution (2 μl of solution A + 1 μl of solution B in 1 ml of DPBS) was added, following 15min incubation. The stained hydrogels were imaged using fluorescent microscope (EVOS M7000, Thermo Fisher Scientific). For this study, three experimental replicates were used. The live (green) and dead (red) stained cells were counted using ImageJ software using atleast 5 images per hydrogel.

### 2.11 Bi-phasic cell-laden hydrogels

To fabricate bi-phasic cell-laden hydrogels, autoclaved PDMS molds (3 mm thickness & 15 mm diameter) with a removable insert blocking half of the mold were used. To start with, HUVECs (P3) and MSCs (P4) were trypsinized, counted (using trypan blue) and were re-suspended in 1:1 ratio with final seeding density of 2.5 × 10^6^ cells ml^−1^. To prepare two different pre-polymer solutions, the cell suspension with same seeding density was centrifuged at 300g for 3mins at 4 °C in two separate 15 ml falcon tubes. For pre-polymer solution 1, supernatant was removed and pre-polymer solution (5% GelMA+0.5% Irgacure 2959 + 2.5nmoles acrydite- or control-aptamer in DPBS) was added and mixed gently by pipetting. Similarly, for pre-polymer solution 2, supernatant was removed and 5% GelMA+0.5% Irgacure 2959 in DPBS solution was added. Afterwards, PDMS molds with an insert blocking half of the area were placed in sterile cell culture petri dish (90 mm diameter) and 200 μl of pre-polymer solution 1 was added. The mold was covered with coverslip and exposed for UV crosslinking (1min, 0.23 mW/cm^2^ intensity). After the crosslinking of one side, the PDMS mold insert and coverslip were carefully removed and pre-polymer solution 2 was added in the rest of the mold followed by UV crosslinking of whole sample (1min, 0.23 mW/cm^2^ intensity). Thereafter, the coverslip and PDMS molds were removed and bi-phasic cell-laden hydrogels were transferred to ultra-low attachment 24-well plate using sterilized spatula. Herein, for VEGF_165_ loading, 1 ml of co-culture medium with VEGF_165_ (10 ng) was added into each sample and incubated at 37 °C for 1hr. Subsequently, the medium was replaced with fresh 1 ml co-culture medium. The medium was changed once every day throughout the experiment. To evaluate the effect of triggered VEGF_165_ release on bi-phasic cell-laden hydrogels, 2.5nmoles CS (1:1 mol ratio of aptamer to CS) into 1 ml co-culture medium was added for acrydite- and control-aptamer based bi-phasic hydrogels on day4 for 24hrs. These samples were analyzed at different time-points. For vessel properties quantification, each sample was categorized into four regions; near aptamer- and near GelMA-side being in immediate vicinity of the interface (≤600 μm; interface at 0 μm), whereas far aptamer- and far GelMA-side were at the far end from the interface (between 2000 to 4000 μm from interface).

### 2.12 Immunostainings

Prior to staining, all of the samples were fixed using 4% formaldehyde solution in DPBS for 30mins, washed with DPBS and permeabilized using 0.1% Triton X-100 in DPBS. Afterwards, the samples were washed with DPBS and blocking solution of 1% FBS in DPBS was added for 45mins. For actin cytoskeleton staining of bi-phasic cell-laden hydrogels, the samples were incubated with Phalloidin-Atto 488 (1:50 in DPBS) or Alexa Fluor-647 Phalloidin (1:40 in DPBS) for 1hr at room temperature, followed by 10mins incubation with Hoechst 33342 (1:2000 in DPBS). For immunostainings, post blocking solution, the samples were washed with DPBS and incubated for overnight at 4 °C with monoclonal anti-actin, α-smooth muscle antibody (mouse, 1:300 in DPBS) and anti-von Willebrand factor antibody (rabbit, 1:200 in DPBS). The samples were then washed with DPBS, IgG (H + L) highly cross-adsorbed goat anti-mouse, Alexa Fluor® 594 (1:1000 in DPBS) and IgG (H + L) cross-adsorbed goat anti-rabbit, Alexa Fluor® 488 (1:1000 in DPBS) solutions were added for 2hrs in dark. Subsequently, the samples were washed with DPBS and incubated with Hoechst 33342 (1:2000) for 10mins in dark. For tight-junctions specific proteins staining, after blocking solution & DPBS washing, the samples were incubated for overnight at 4 °C with human VE-Cadherin antibody (mouse, 1:200 in DPBS). The samples were washed with DPBS and incubated with IgG (H + L) cross-adsorbed goat anti-mouse, Alexa Fluor® 488 (1:1000 in DPBS) for 2hrs in dark. After 1hr, Phalloidin 647 (1:40 in DPBS) was added into the secondary antibody staining solution and allowed to incubate for 1hr in dark. Thereafter, the samples were washed and Hoechst 33342 (1:2000) was added for 10mins in dark. Once stained, samples were washed with DPBS, 500 μl DPBS was added and stored at 4 °C until imaged.

### 2.13 Statistical analysis

Statistical analysis was performed using GraphPad Prism 7 software. Two-way analysis of variance (ANOVA) with Tukey's multiple comparisons test was used to analyze the data. Significance was set at p < 0.05. The data is represented as mean ± SD.

## 3 Results and discussion

### 3.1 Acrydite-modified aptamers enhance aptamer retention and CS recognition within aptamer-functionalized hydrogels

**Fig. 1:**
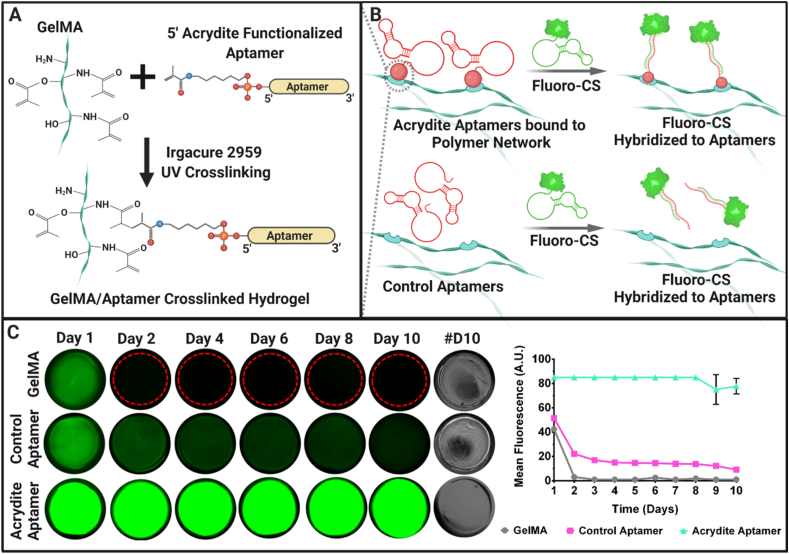
The concept of aptamer-functionalized hydrogels and aptamer retention within gelatin methacryloyl (GelMA) hydrogels. (A) Schematic representation of photo-crosslinked GelMA with 5′acrydite-modified VEGF_165_ specific aptamer sequence, in the presence of photoinitiator (Irgacure 2959) and UV light. (B) Schematics explaining the concept of 5′acrydite-modified aptamers being covalently linked into GelMA network, whereas, due to the absence of 5′acrydite group (red circle), the control-aptamers are freely diffusible within the polymer network. Due to molecular recognition, upon addition of 5′-fluorescently labelled complementary sequence (Fluoro-CS) in the 5′acrydite-aptamer-functionalized hydrogels (Acrydite Aptamers) and control-aptamer-functionalized hydrogels (Control Aptamers), the Fluoro–CS–aptamer hybridization occurs. (C) The fluorescence microscopic images of the GelMA, “Control Aptamer” and “Acrydite Aptamer” -functionalized hydrogels after 24hrs incubation with Fluoro-CS at 37 °C. #D10 indicates the brightfield images of hydrogels on day10. The fluorescence intensity corresponding to the presence of Fluoro-CS indicates the presence of aptamers within the hydrogels at various time-points. The red dotted line in GelMA hydrogels from day2 onwards shows the hydrogel border. The mean fluorescence of all samples over different time-points have been shown in the graph. The experiment was performed with n = 3 experimental replicates and values are represented as mean ± SD.

To develop the desired versatile platform, we synthesized photocrosslinked aptamer-functionalized gelatin methacryloyl (GelMA) hydrogels via free-radical polymerization initiated with UV light exposure **(**Fig. 1A**)**. The oligonucleotide sequence specific for VEGF_165_ [30,31], was utilized in both unmodified form (hereafter referred as “control-aptamer”) and 5′-acrydite modified form (hereafter referred as “acrydite-aptamer”) (Fig. S1 and Table S1). Upon initiating the free-radical polymerization, the unsaturated bonds present in acrydite groups enable covalent aptamer incorporation within the polymer network. [32,33]. The methacrylation degree and monomer concentration are critical parameters governing the polymer crosslinking density, that in-turn affects the hydrogel porosity, its pore size and mechanical properties. [34,35]. Specifically, GelMA hydrogels with high methacrylation degree have often showed low porosity and high elastic modulus. [34]. As high porosity supports mass transport to cells and suitable pore size allows cell ingrowth, both of which are favorable conditions for 3D culture [34], GelMA with a medium methacrylation degree (∼60%) was utilized for the fabrication of aptamer-functionalized hydrogels throughout the study (Fig. S2).

To demonstrate the versatility of the platform, we first set out to verify aptamer retention and their ability for CS recognition within the hydrogels by using 5′-Alexa Fluor 488 fluorophore labelled CS (Fluoro-CS) at physiological temperature (i.e., 37 °C) (Fig. 1B and C). The acrydite-aptamer-functionalized hydrogels exhibited higher fluorescence throughout the study, confirming stable aptamer incorporation into the hydrogel network (Fig. 1C). The control-aptamer-functionalized hydrogels showed lower fluorescence after the initial 24hrs, followed by gradual decrease of the fluorescent signal over time. Without acrydite modification, the control-aptamers tend to physically entrap within polymer network during polymerization, which enables gradual aptamer diffusion out of the polymer network. As expected, the plain GelMA hydrogels (without aptamer) showed no detectable fluorescence after 48hrs (Fig. 1C). Furthermore, to evaluate the presence of the acrydite-aptamer and Fluoro-CS diffusion throughout the acrydite-aptamers hydrogel thickness, bi-phasic hydrogels (having one side with acrydite-aptamer and one side without aptamer; 3 mm thickness & 15 mm diameter) were imaged using confocal microscopy after 24hrs incubation with Fluoro-CS at 37 °C. Three-dimensional projections of confocal z-stacks confirms the CS-Fluoro penetration (z = 240 μm) through the hydrogel thickness while a clear interface with GelMA region (in blue color) is maintained (Fig. S3 & Video S1). Combined, these observations suggest that Fluoro-CS was able to penetrate within the (acrydite- & control-) aptamer-functionalized hydrogels at macroscopic scale within a timeframe of 24hr incubation (irrespective of aptamer being covalently-bound or physically entrapped) and displayed high molecular recognition via aptamer-Fluoro-CS hybridization at physiological temperature.

### 3.2 Aptamers crosslinked within hydrogel matrix mediates maximum VEGF_165_ sequestration and programmable release via CS hybridization

**Fig. 2:**
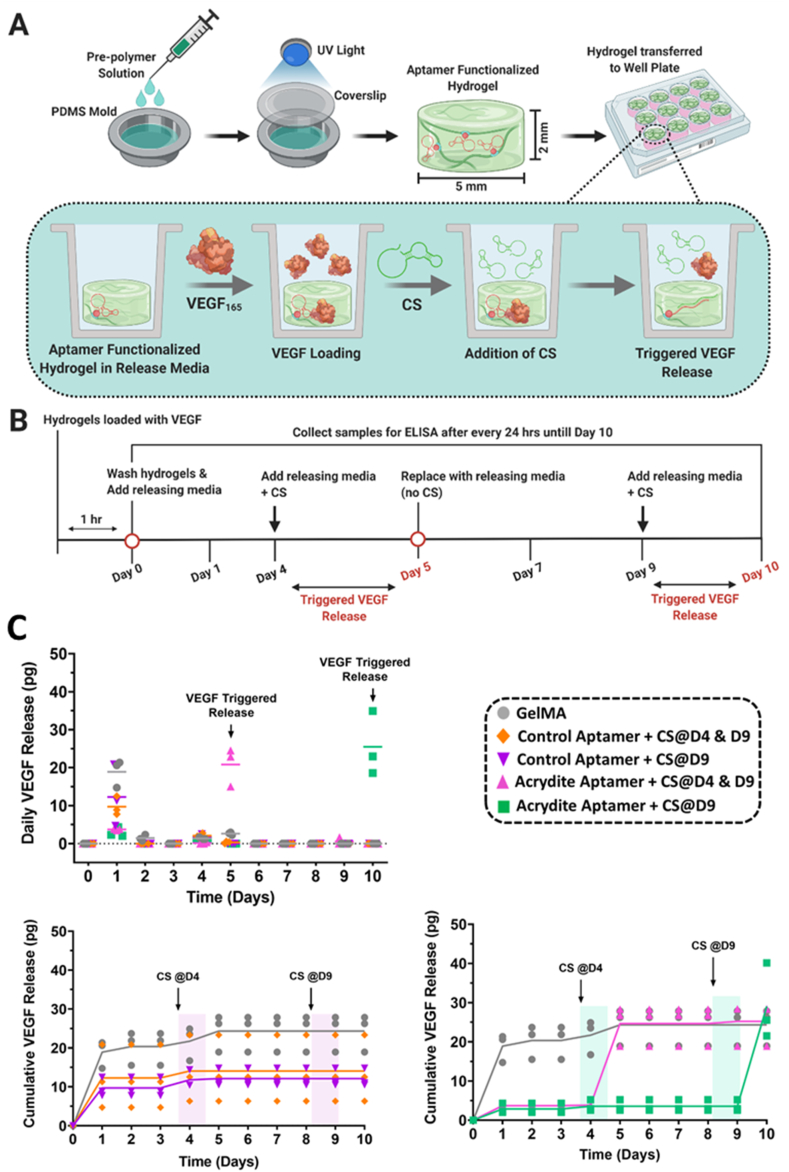
Temporally controlled VEGF_165_ release from the aptamer-functionalized hydrogels. (A) Schematic representation of the aptamer-functionalized hydrogel fabrication, its ability to sequester VEGF_165_ from the release media via affinity interactions and on-demand triggered release of VEGF_165_ in presence of complementary sequences (CS). (B) The complete timeline of the experiment. The aptamer amount used was 2.5 nmoles (25 μM) and initial VEGF_165_ loading amount was 10 ng. (C) The daily (top) and cumulative (bottom) VEGF_165_ release from control-aptamer- and acrydite-aptamer-functionalized hydrogels over 10 days in presence or absence of CS. To trigger the first round of VEGF_165_ release on day5, the CS was added to specified hydrogels on day4 for 24hrs (as indicated in figure). Furthermore, to trigger the second round of VEGF_165_ release, the CS was added to all aptamer hydrogel samples on day9, except for GelMA hydrogels. The mole ratio of CS to aptamers used was 1:1. The graphs are represented as mean with individual data points. The experiment was performed with three experimental replicates, n = 3.

Having verified the aptamer retention and its ability for CS hybridization within aptamer-functionalized hydrogels, we next set out to validate their functionality for VEGF_165_ sequestration and programmable, triggered VEGF_165_ release via aptamer-CS hybridization. Firstly, for verifying the rapid VEGF_165_ sequestration ability and subsequent GF release over time, the hydrogels were incubated with VEGF_165_ (10 ng) containing loading medium for 1hr (Fig. 2A and B). The results indicated higher VEGF_165_ sequestration within aptamer-functionalized hydrogels even after 1hr incubation (50% and 46% for control-aptamer and acrydite-aptamer, respectively) compared to GelMA hydrogel (33%) (Fig. S4). To further visualize the differential sequestration of VEGF_165_ within hydrogels, bi-phasic hydrogels (having one side with acrydite-aptamer and one side without aptamer; 3 mm thickness & 15 mm diameter) loaded with VEGF_165_ for 1hr were immunostained using anti-VEGF antibody. The confocal microscopic images showed increased levels of VEGF_165_ sequestration (in red color) within acrydite-aptamer region compared to the GelMA region (in blue color) (Fig. S5). Gelatin-based hydrogels display inherent GF sequestration properties because of their electrostatic interactions with oppositely charged GFs like VEGF, which can explain the relatively high sequestration for GelMA hydrogels. As expected, the increased sequestration in aptamer-functionalized hydrogels is likely due to the binding affinity of GF with aptamer. The insignificant difference in VEGF_165_ sequestration among aptamer-functionalized hydrogels confirm the similar GF sequestration ability of the used aptamer, irrespective of being covalently crosslinked or freely diffusible within the polymeric network. However, as shown in Fig. 1C, the freely diffusible behavior of control-aptamer becomes prevalent within the 24hrs of incubation that could result into the initial burst release of VEGF_165_. Notably, the present GF loading route decouples the loading process from the hydrogel fabrication process, thus providing a clear advantage over conventional GF loading strategies, where free-radicals generated during hydrogel preparation could potentially harm the GF bioactivity. In a tissue engineering context, the present approach provides great flexibility over GF loading at different time-points after hydrogel fabrication, which is adaptable according to the needs of developing engineered tissue.

To explore the dynamic nature of this platform, we next examined the triggered GF release behavior from aptamer-functionalized hydrogels by supplementing CS (1:1 aptamer to CS mole ratio) into the releasing medium at different time-points (Fig. 2C). As expected, the control-aptamer-functionalized and GelMA hydrogels showed high burst release of VEGF_165_ on day1 (10.99 pg and 18.89 pg, respectively) and near zero release from day2 onwards. In contrast, acrydite-aptamer-functionalized hydrogels exhibited minimal initial release on day1 (3.30 pg), confirming significantly reduced initial burst release and efficient VEGF_165_ sequestration within the hydrogel. Upon the first round of triggered release with CS addition on day4, the acrydite-aptamer-functionalized hydrogels showed high VEGF_165_ release within 24hr (20.81 pg), compared to GelMA and control-aptamer-functionalized hydrogels (2.62 pg and 0.32 pg, respectively). For testing the platform's functionality at later time-point, the second triggered release with CS addition on day9 was attempted with all samples. Interestingly, the acrydite-aptamer- and control-aptamer-functionalized hydrogels that were already triggered on day4 showed no additional release, but acrydite-aptamer-functionalized hydrogel triggered only on day9 showed high VEGF_165_ release on day10 (25 pg) (Fig. 2C). This indicates that CS used in present study was able to regulate the binding functionality of aptamers and induce rapid dissociation of aptamer-protein complexes to trigger VEGF_165_ release within acrydite-aptamer samples. The used aptamer to CS mole ratio (1:1) showed a maximum release of 25 pg at once, followed by no subsequent release upon second CS triggering indicating its saturation. However, it is speculated that the triggered VEGF_165_ release can be modulated by varying triggering time and CS mole ratio. This proof-of-concept data shows the rapid sequestration (within 1hr) and on-demand VEGF_165_ releasing capability of acrydite-aptamer-functionalized hydrogels. The cumulative VEGF_165_ release data confirms overall higher release from acrydite-aptamer-functionalized and GelMA hydrogels (25–29 pg), than control-aptamer-functionalized hydrogels (12–14 pg) (Fig. 2C). Considering control-aptamers not being covalently crosslinked within the polymeric network, as confirmed by retention data (Fig. 1C), a portion of VEGF_165_ molecules diffusing into the releasing medium is likely to be bounded with control-aptamers via affinity interaction. Upon investigating the sensitivity of the used ELISA assay for detecting VEGF_165_ molecules complemented with control-aptamers, lower signals (with overall difference of 28%) were detected for VEGF_165_ incubated with control-aptamers compared to VEGF_165_ in DPBS (Fig. S6). It is anticipated that this disparity in ELISA sensitivity towards VEGF_165_ molecules in the presence of control-aptamers, could be a possible reason for the overall lower VEGF_165_ release values from control-aptamer-functionalized hydrogels. It should further be noted that a cumulative release of approximately 30 pg constitutes only a small fraction of the 10 ng that was loaded per sample, from which up to half was sequestered into the sample as determined by analysis of the remaining VEGF_165_ in the loading fluid. Even though this could indicate that the major fraction of loaded VEGF_165_ remains in the samples after 10 days of release, this is unlikely given the low level of staining for VEGF_165_ in the biphasic system in Fig. S5. If the binding characteristics of unmodified GelMA would be strong enough to retain the major fraction of VEGF_165_ over the course of 10 days, the differential effect of binding to the aptamer would be small, resulting in only a small or undetectable difference in the staining. It remains unclear what has caused this discrepancy, but as the VEGF_165_ sequestration and release experiments were performed separately, variations among individual samples used in the experiments cannot be ruled out. It is speculated that by modulating aptamer's binding affinity and mole ratio, an improved GF retention capacity can be achieved. Aptamer-functionalized superporous hydrogels with high binding affinity have been previously shown to retain up to 90% of the GF (PDGF-BB) after 24hr incubation with washing solution, compared to the non-functionalized (14%), low binding affinity (28%) and medium binding affinity aptamer-functionalized hydrogels (63%), respectively. [33]. Similarly, higher GF retention was reported with increased mole ratio of aptamer to GF such as 53% (1:1 mol ratio), 90% (10:1 mol ratio) and 96% (100:1 mol ratio), respectively. [33]. In the previously reported study, small volumes of highly concentrated GF solution were directly loaded onto partially dehydrated superporous hydrogels which were then incubated with the GF solution for 24hr. [33]. The present study on the other hand employs GF sequestration from a large volume of medium supplemented with a more physiological concentration of GF during 1hr of incubation. Even though the previously employed approach is likely to result in more efficient GF loading of the aptamer functionalized hydrogel, the presented approach is better compatible with patterned hydrogels or potential applications where the GF is sequestered *in vivo*. Taken together, these results confirmed acrydite-aptamer functionalized hydrogel's potential for rapid (within 1hr loading) and localized (confined within aptamer region) VEGF_165_ sequestration from the culture medium as well as their capability for triggering VEGF_165_ release via aptamer-CS hybridization.

### 3.3 Aptamer incorporation influences physicochemical properties of aptamer-functionalized hydrogels

**Fig. 3:**
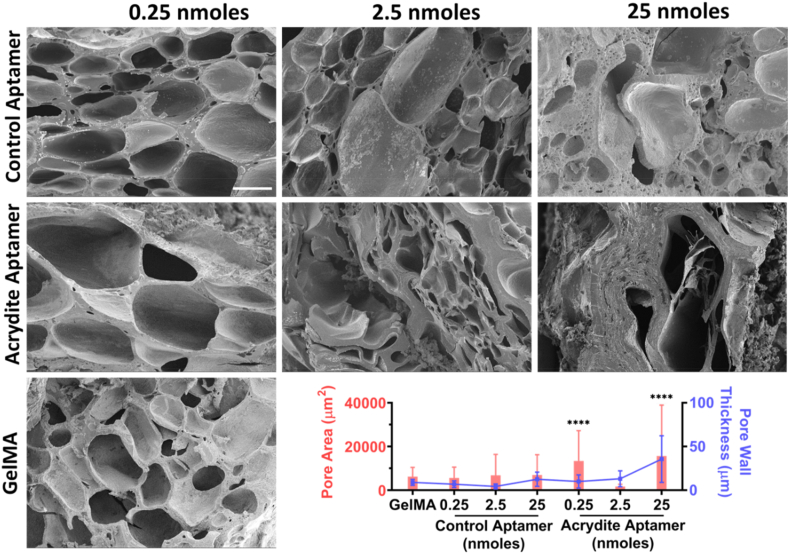
Pore properties of aptamer-functionalized hydrogels with various aptamer amounts. SEM images of the cross-section of acrydite-aptamer- and control-aptamer-functionalized hydrogels with different aptamer amounts, namely 0.25 nmoles, 2.5 nmoles and 25 nmoles aptamers per sample. The GelMA hydrogels without aptamers were also used. Scale bar is 100 μm and magnification is 200x. Pore area and pore wall thickness of different hydrogel samples quantified by ImageJ using the representative SEM images. The data is represented as mean ± S.D. The statistical significance among pore area and pore wall thickness within each sample type was calculated using two-way ANOVA with Tukey's multiple comparisons test where **** means p < 0.0001.

To understand the effect of aptamer incorporation in GelMA hydrogels on their overall physicochemical properties, aptamer-functionalized hydrogels with varying aptamer amounts were assessed (Fig. 3). The scanning electron micrographs (SEM) data of acrydite-aptamer-functionalized hydrogels showed significant aptamer amount dependent pore properties. For example, low aptamer (0.25nmoles) resulted in an increase of the average pore area compared to GelMA samples (13,320 μm^2^ compared to 6079 μm^2^ respectively). An increase in the concentration of acrydite-aptamer resulted in denser polymeric crosslinking, which is evident by increased wall thickness and decreased pore size for 2.5nmoles samples (∼13 μm and 1,462 μm^2^) and collapsing pores in 25nmoles samples accompanying higher wall thickness and larger remaining pores (∼35 μm and 15,551 μm^2^). Control-aptamer-functionalized hydrogels on the other hand showed similar pore properties as GelMA hydrogels for all tested aptamer amounts (for example, an average pore area within 6000–6,900 μm^2^ range). It is noteworthy that pore size determined from SEM images of lyophilized hydrogels, does not represent the hydrogel ultrastructure in a hydrated state that is experienced by encapsulated cells. [36]. Instead, the observed porosity is a result of the freezing-lyophilization process and represents the relative density of the hydrated hydrogels for comparison.

**Fig. 4:**
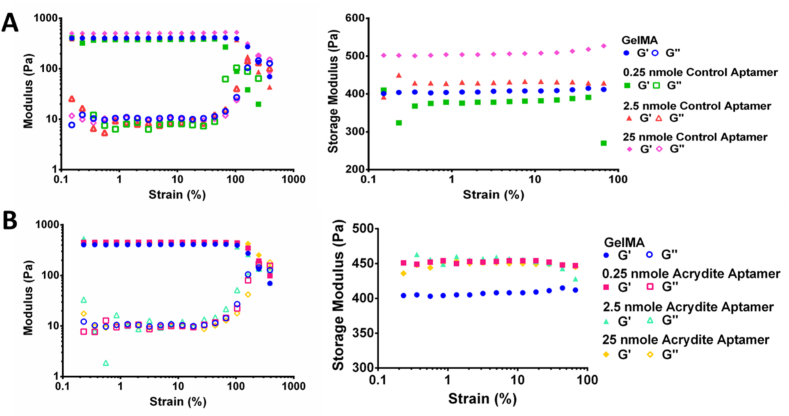
Rheological analysis of aptamer-functionalized hydrogels with various aptamer amounts, namely 0.25 nmoles, 2.5 nmoles and 25 nmoles aptamers per sample. (A) control-aptamer-functionalized and (B) acrydite-aptamer-functionalized hydrogels, along with GelMA hydrogels. The storage modulus (filled symbols) and loss modulus (empty symbols) of the respective hydrogels at fixed angular frequency of 1 rad/s are shown.

In correlation with SEM data, the rheology results revealed higher storage modulus for acrydite-aptamer-functionalized hydrogels (∼450Pa) compared to GelMA hydrogel (404Pa) (Fig. 4). This effect was independent of the aptamer amount present within the hydrogel. The control-aptamer-functionalized hydrogels exhibited a slightly lower storage modulus for the lowest tested aptamer amount (∼379Pa for 0.25nmoles). The storage modulus increased upon further increasing the aptamer amounts (∼430Pa for 2.5nmoles; ∼509Pa for 25nmoles). While the exact reason for these changes in mechanical properties of the hydrogels is unclear, one possibility may be linked to the hydrophilic nature of aptamers resulting in additional swelling of hydrogels when present in higher amounts, and thus contributing towards a higher storage modulus. [37]. This behavior could be anticipated for control-aptamers that are physically entrapped within hydrogel network, whereas covalently crosslinked acrydite-aptamers in the amounts used in this study are less likely to be available for participating in swelling, mainly because of the steric hindrances caused due to the close proximity of covalently immobilized acrydite-aptamers with GelMA polymeric network. [38].

### 3.4 Bioactivity of acrydite-aptamer functionalized cell-laden hydrogels post-VEGF_165_ sequestration and its triggered release via CS hybridization

**Fig. 5:**
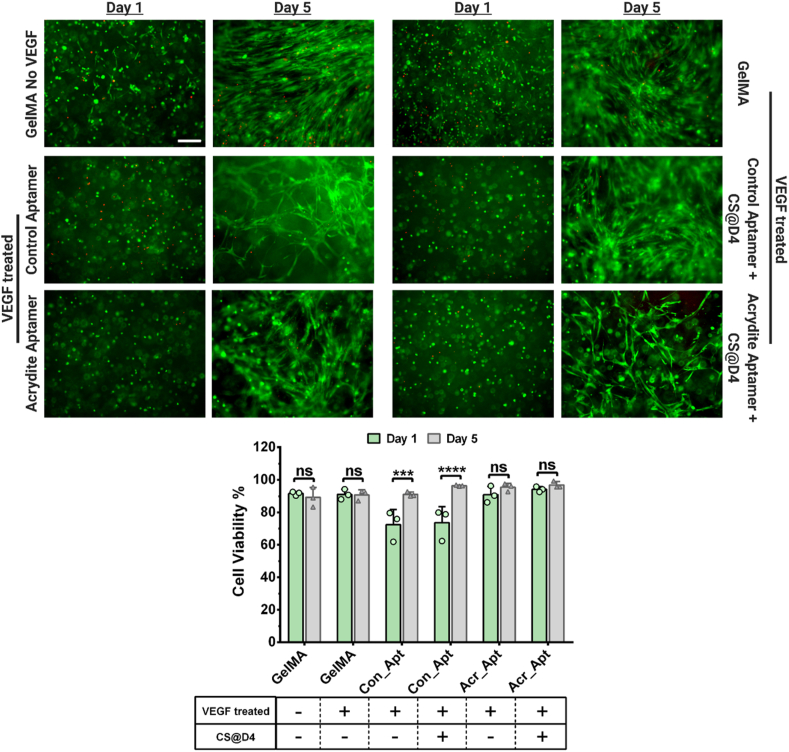
Cell viability of the 3D co-cultured aptamer-functionalized hydrogels with or without VEGF_165_ loading. Live/Dead stained fluorescent microscopic images of 3D cultured hydrogels with control-/acrydite-aptamer-functionalized hydrogels in the presence or absence of CS addition on day4, on different time points. The HUVECs and MSCs were co-cultured in 1:1 ratio. For VEGF_165_ loading, the hydrogels were incubated with co-culture medium (without VEGF_165_ supplement) added with known VEGF_165_ amount (10 ng), for 1hr at 37 °C. Plain GelMA hydrogels, with or without 1hr VEGF_165_ loading were also studied. The scale bar is 200 μm. Cell viability % quantification was performed using ImageJ software. The quantification was performed with three experimental replicates, n = 3. The statistical significance was calculated using two-way ANOVA with Bonferroni's multiple comparisons test where ***p = 0.0005, ****p < 0.0001 and ns stands for not significant.

ECM is a highly dynamic microenvironment consisting of multifunctional components (for example, proteoglycans and glycoproteins) that are capable of promoting cell adhesion as well as controlling GF presentation to cells by sequestering soluble GFs and releasing them via enzymatic degradation. Both soluble and ECM-bound GFs facilitate the spatiotemporal regulation of cellular responses. While many strategies have been developed to recapitulate the critical complexities of native ECM, the use of CS for triggering GF release, along with unique properties of aptamers and GelMA, provide this platform with the ability to precisely tune GF bioavailability within hydrogel microenvironment to probe and direct encapsulated cell responses in 4 dimension (4D). To this end, we next demonstrated the bioactivity of the platform throughout the process of VEGF_165_ loading and triggered CS mediated release; co-cultures of human umbilical vein endothelial cells (HUVECs) and human mesenchymal stromal cells (MSCs) were used as model cell systems and were subjected to 3D cell encapsulation for fabricating cell-laden aptamer-functionalized hydrogels. These proof-of-concept experiments were designed to incubate cell-laden samples with VEGF_165_ in co-culture medium for 1hr to enable rapid GF sequestration, followed by washing and medium replacement. Additionally, VEGF_165_ release was triggered by CS addition into the culture medium for 24hrs on day4 of culture. To ensure that the observed cell behavior is mainly due to the bioavailability of externally loaded GF, no VEGF supplements were added into the co-culture medium after the initial GF loading step. Regardless of CS addition, high cell viability (>90%) in acrydite-aptamer-functionalized and GelMA hydrogels on day1 and day5 were observed (Fig. 5). However, in control-aptamer-functionalized hydrogels relatively lower cell viability (>70%) on day1 was observed that significantly increased (>90%) by day5, irrespective of CS addition. The initial lower viability in control-aptamer samples remains unclear, but could be related to the cells preference for matrix bound GFs as opposed to soluble ones. [39].

### 3.5 Spatially patterned aptamers within bi-phasic hydrogels selectively guide cell behavior within 3D microenvironment

**Fig. 6:**
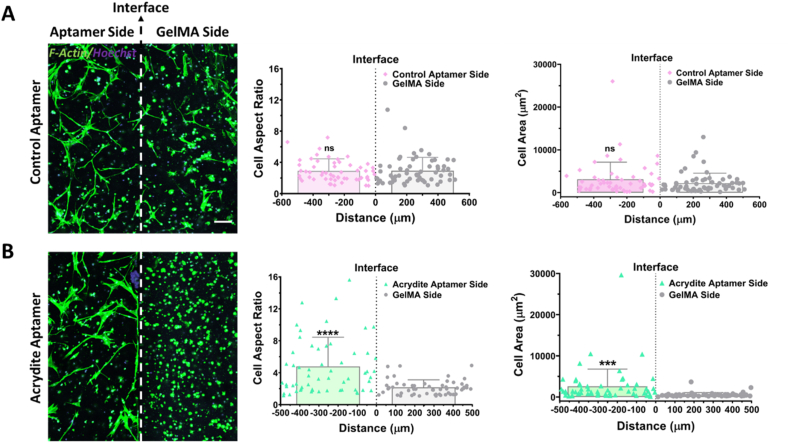
Bi-phasic cell-laden acrydite-aptamer-functionalized hydrogels generate selectively guided cell responses on day3. The maximum projection confocal images of the HUVECs and MSCs, co-cultured within VEGF_165_ loaded (A) control-aptamer- and (B) acrydite-aptamer-functionalized bi-phasic hydrogels. For the ease of understanding, each bi-phasic hydrogel was categorized into two regions; aptamer side and GelMA side where the white dashed line indicates the interface. The cells were stained with cytoskeletal F-actin Phalloidin (green) and Hoechst (blue). The scale bar is 100 μm. Quantification of the individual cell area plotted against the distance from the interface within the (A) control-aptamer- and (B) acrydite-aptamer-functionalized bi-phasic hydrogels. The quantification was performed using ImageJ software, where values are represented in scatter plot with individual data points along with overall mean ± S.D. The calculations were performed with three technical replicates, n = 3. The statistical significance between aptamer and GelMA sides were calculated using unpaired two-tailed *t*-test with Welch's correction where ***p = 0.0004, ****p < 0.0001 and ns stands for not significant.

Following the successful bioactivity confirmation of cell-laden acrydite-aptamer-functionalized hydrogels, we next aimed to demonstrate their fundamentally unique capabilities compared to the existing GF releasing strategies. We hypothesized that by spatially controlling local GF bioavailability using this platform, we could selectively guide cell behavior in 3D microenvironment. To test this, we designed bi-phasic cell-laden hydrogels with (control- or acrydite-) aptamer-functionalized hydrogels on one-side and plain GelMA on the other side via two-step photocrosslinking. These bi-phasic hydrogels were subsequently loaded with VEGF_165_ (10 ng) for 1hr. Microscopy data of F-actin stained samples confirmed the ability of the encapsulated cells to sense the localized presence of acrydite-aptamer-bound-VEGF_165_ within the hydrogels, resulting in increased cell adherence and spreading within the aptamer regions on day3 (Fig. 6). For quantification, the images with F-actin staining were processed via thresholding, binary and watershed segmentation tools in ImageJ software. The results indicates higher average cell area (2569 μm^2^) and aspect ratios in acrydite-aptamer side than the GelMA side (542 μm^2^). In control-aptamer samples, both sides displayed similar cell responses (average cell area–3133μm^2^ & 2220 μm^2^; aspect ratio–2.8 & 2.9). This limited effect in the control-aptamer samples is likely to be attributed to the freely diffusible nature of control-aptamer-bound-VEGF_165_ molecules within the hydrogel matrix, correlating with our previous aptamer retention (Fig. 1C) and VEGF quantification data (Fig. 2C). Due to the bi-phasic nature of the samples, with both sides being exposed to the same pool of culture medium, it is likely that control-aptamer-bound-VEGF_165_ molecules diffuse out of the aptamer hydrogel region, thus being accessible to cells encapsulated within both regions. On the other hand, as acrydite-aptamers are covalently immobilized, this confines the acrydite-aptamer-bound-VEGF_165_ bioavailability within one region. When comparing the aptamer containing regions of control-aptamer samples with acrydite-aptamer samples, higher cell aspect ratios are seen in the acrydite-aptamer samples. Even though the exact mechanisms behind this are unclear, the increased cellular response within acrydite-aptamer regions could be attributed to the ability of matrix-bound VEGF to elicit more pronounced cell responses compared to soluble VEGF. [39]. It has previously been shown that matrix-bound VEGF induces its receptor (VEGFR2)-β1-integrin complex formation and increases the β1-integrin targeting to focal adhesions. [39].

### 3.6 Spatiotemporally controlled VEGF_165_ bioavailability within acrydite-aptamer bi-phasic hydrogels manipulates microvascular network organization in 3D microenvironment

**Fig. 7:**
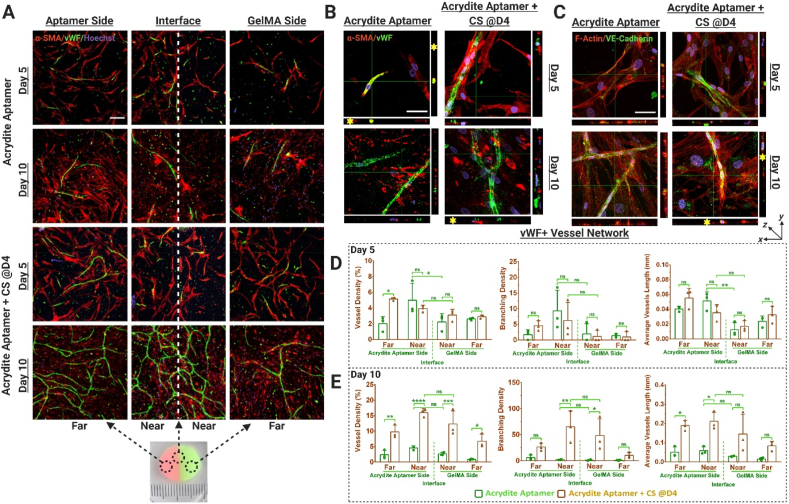
Spatiotemporally controlled vascular network organization within acrydite-aptamer-functionalized bi-phasic cell-laden hydrogels. (A) Immunostained maximum projection confocal images showing von Willebrand factor (vWF) expression (green) as a marker for endothelial cells and α-smooth muscle actin (α-SMA) (red) as a marker for MSCs differentiation to mural cells, within the HUVECs and MSCs co-cultured, bi-phasic hydrogels (with or without CS treatment on day4). The representative photographic image of the actual bi-phasic hydrogel, showing two distinct sides (one with aptamer and other without) with a distinct interface. Considering its big size, each hydrogel was categorized into four regions; near aptamer- and near GelMA-side being in immediate vicinity of the interface, whereas far aptamer- and far GelMA-side were at the far end from the interface. Scale bar is 200 μm. Orthogonal views of the confocal z-stacks showing (B) vWF+α-SMA + Hoechst stained samples & (C) F-actin Phalloidin + VE-Cadherin + Hoechst stained samples, at higher magnification, exhibits the developing vascular networks. At the cross-section of the developing vessel, a round lumen like vascular structure could be observed in orthogonal view (indicated by yellow *). The scale bar is 50 μm. Quantification of vWF + stained vessel network in these samples using Angiotool Software on day5 (D) and day10 (E). The values are represented as mean ± SD, along with individual data points. The calculations were performed with three technical replicates, n = 3. The statistical significance was calculated using two-way ANOVA with tukey's post-hoc test where *p < 0.05, **p < 0.01, ***p < 0.001, ****p < 0.0001 and ns stands for not significant.

**Fig. 8:**
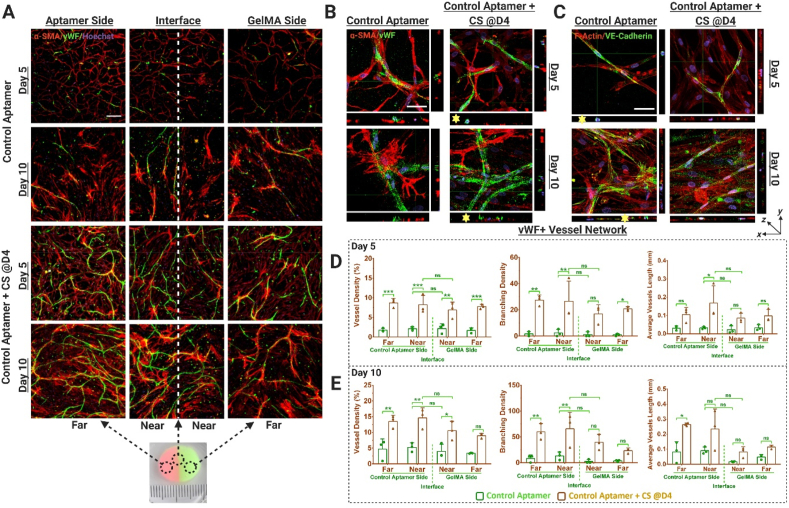
Spatiotemporally controlled vascular network organization within control-aptamer-functionalized bi-phasic cell-laden hydrogels. (A) Immunostained maximum projection confocal images showing von Willebrand factor (vWF) expression (green) as a marker for endothelial cells and α-smooth muscle actin (α-SMA) (red) as a marker for MSCs differentiation to mural cells, within the HUVECs and MSCs co-cultured, bi-phasic hydrogels (with or without CS treatment on day4). The representative photographic image of the actual bi-phasic hydrogel, showing two distinct sides (one with aptamer and other one without) with a distinct interface. Considering its big size, each hydrogel was categorized into four regions; near aptamer- and near GelMA-side being in immediate vicinity of the interface, whereas far aptamer- and far GelMA-side were at the far end from the interface. Scale bar is 200 μm. Orthogonal views of the confocal z-stacks showing (B) vWF + α-SMA stained samples & (C) F-actin Phalloidin + VE-Cadherin stained samples, at higher magnification, showing the developing vascular networks. At the cross-section of the developing vessel, a round lumen like vascular structure could be observed in orthogonal view. The scale bar is 50 μm. Quantification of vWF + stained vessel network in these samples using Angiotool Software on day5 (D) and day10 (E). The values are represented as mean ± SD, along with individual data points. The calculations were performed with three technical replicates, n = 3. The statistical significance was calculated using two-way ANOVA with tukey's post-hoc test were *p < 0.05, **p < 0.01, ***p < 0.001, ****p < 0.0001 and ns means not significant.

VEGF plays a vital role in early regulation of angiogenesis. Spatiotemporally controlling its bioavailability within engineered matrices (hydrogel) could be a valuable tool to direct the tubular morphogenesis of endothelial cells for achieving mature and stable vascular networks. Having established the effect of aptamer-functionalized bi-phasic hydrogels on cellular adherence and spreading, we next investigated its capacity for translating this effect with temporal control in guiding vascular morphogenesis. To do so, the bi-phasic samples were triggered with CS for VEGF_165_ release at different time-points and the effect of this on microvascular network organization within biphasic samples was studied. The microscopy data confirms positive expression of von Willebrand factor (vWF, endothelial cell specific marker) and α-smooth muscle actin (α-SMA, smooth muscle cells specific marker)(Fig. 7, Fig. 8). In agreement with previous research, the interaction between endothelial cells and MSCs in co-cultures directs MSCs differentiation towards the smooth muscle cell lineage, indicating vascular network stabilization and maturation. [40]. Furthermore, the microscopic images in orthogonal view revealed the presence of circular/elliptical lumen-like structures within vWF + networks wrapped by α-SMA + cells, mimicking the early tubular morphogenesis of endothelial cells in native tissues (Fig. 7, Fig. 8B & S7 and Video S2). VE-cadherin, which is an endothelial intercellular junctional protein, plays a substantial role in endothelial cell tubular morphogenesis, vascular stability and maturation. The positive expression of VE-cadherin in the developing vascular networks is an early marker for vessel stabilization and maturation. As expected, VE-cadherin expression increased over time (Figs. 7C and 8C).

Quantification of vascular organization revealed that triggered VEGF_165_ release (via CS addition) could spatiotemporally regulate vessel properties within acrydite-aptamer and GelMA regions (Fig. 7A,D&E) (Fig. S8, video S3). Upon comparing the network parameters among acrydite-aptamer samples treated with/without CS, the temporal control over vascular network organization triggered via CS addition becomes apparent. For instance, samples without CS addition showed higher vessel density in near aptamer region (region close to the interface on aptamer side; 4.99%) compared to near GelMA (region close to the interface on GelMA side; 2.21%) (p = 0.0421). Herein, the vessel density is defined as the percentage of area occupied by vWF + vessels within the total area (i.e., %vessels/total area). However, after 24hr CS treatment, this difference became insignificant (Fig. 7D). Even though the vessel density became indistinguishable across the interface by day10 (independent of CS addition), pronounced vessel density was observed specifically in near aptamer region for samples treated with CS (15.94%) compared to samples without CS (4.57%) (p < 0.001) (Fig. 7E). Similar trends were observed in near GelMA regions with higher vessel density in CS treated samples (12.30%) compared to samples that were not treated with CS (2.61%) (p = 0.004) by day10 (Fig. 7E). Evaluation of other vessel parameters such as branching density (i.e., number of vessel junctions/area) and average vessel length (mean length of all the vessels in image) corroborated this trend. On day5, lower average vessel length in near aptamer regions were observed for samples without CS treatment (0.0513 mm) compared to samples with CS (0.035 mm), but by day10, samples with CS treatment showed a pronounced increase in average vessel length (0.211 mm) compared to samples without CS (0.058 mm) (Fig. 7D&E). As expected, in control-aptamer samples significantly increased vessel network formation was observed when comparing CS treated samples with samples without CS on day5 and day10 (Fig. S9 & video S4). However, no significant differences were observed across the interface in all samples (Fig. 8A,D&E); owing to the diffusible nature of control-aptamer-bound-VEGF_165_ molecules. Furthermore, significant differences in vascular organization were observed across different spatial regions of acrydite-aptamer samples on day5, confirming its spatial control. For instance, the acrydite-aptamer samples without CS addition on day5 showed significantly higher vessel density in near aptamer region (4.995%) than near-GelMA region (2.215%) (p = 0.0421). Similar trend was observed for average vessel length across the interface (0.0513 mm, near-aptamer & 0.0124 mm, near-GelMA) (p = 0.0035) (Fig. 7D). It is noteworthy that the effect of aptamer's spatial patterning within bi-phasic hydrogels is pronounced at the interface (near aptamer/near GelMA regions) (Fig. 6) and the far-regions on both sides behave as bulk hydrogels. It is evident from Fig. 6, Fig. 7 that the acrydite-aptamer's spatial patterning across the interface influences cellular responses till day 5 of culture and tends to decrease by day10, leading to insignificant difference in vessel properties across the interface. Additionally, insignificant differences in vessel and branching densities were observed upon comparing control-aptamer to acrydite-aptamer samples within near- and far-aptamer regions on day5 and day10 (Fig. S10). Similar trends were observed among same samples in the presence of CS on day5 and day10. Interestingly, significant differences in average vessels length was observed among control-aptamer and acrydite-aptamer samples at both time-points within near-aptamer regions (near to interface on aptamer side) of the bi-phasic hydrogels. However, upon CS addition no significant differences were observed. The overall increased vessel properties in CS-treated samples (control- & acrydite-aptamer) at different time-points (day5, 10), confirms the higher bioactivity of the released GF via CS hybridization and spatially controlled vascular organization in acrydite-aptamer samples across the interface by day5, shows the platform's potential for regulating vascular morphogenesis *in vitro* by spatiotemporally controlling GF bioavailability.

The establishment of optimally organized vascular networks is currently a major hurdle for clinical translation of engineered tissues at large-scale. While many strategies have previously shown success with pre-vascularized scaffolds or cell patterning, they often overlook to account for vascular remodeling, thus hampering the functionality of these networks. [2]. Given the mobile nature of encapsulated endothelial cells within scaffolds, the initially developed vascular networks *in vitro* tend to remodel after implantation. At this stage, using platforms that provide additional cues to guide the vascular remodeling processes, can enable the sustainment of well-organized vascular networks for longer durations. The cell culture data in this study confirm the platform's ability to not only form lumen-like microvascular networks within a hydrogel matrix, but also to enable spatiotemporally controlled network organization. These unique properties could possibly help to better control vascular network remodeling processes *in vivo*.

In addition to demonstrating their ability for selectively directing cell responses by controlling GF bioavailability, this study also establishes that (i) the acrydite-aptamer-functionalized hydrogels were stable, exhibiting minimum VEGF_165_ leakage and (ii) the hydrogels display high bioactivity of sequestered VEGF_165_ *in situ*, for at least ten days in culture. Although GelMA degradation could be a limiting factor affecting GF release in studies involving longer culture periods, this could be addressed by modulating polymer concentration, methacrylation degree and crosslinking density. [41]. GelMA being the polymeric backbone, makes this a versatile bioactive platform. GelMA hydrogels possess cell adhesion properties, owing to RGD motifs from gelatin and allow for *in vitro* enzymatic degradation, indicated by the presence of matrix metalloproteinase (MMP)-sensitive motifs suitable for ECM remodeling. [41]. Moreover, GelMA is compatible with various biofabrication techniques (such as, 3D bioprinting, photo-patterning, etc.), that ensures adaptability of the platform for creating tissue-specific 3D structures and patterns with controlled cellular behavior in 4D. While in this study spatial patterning is limited to a relatively large biphasic disk, 3D bioprinting and photopatterning will also result in more defined spatial patterns of aptamer availability, enabling a more precise investigation of the spatial control over vascular organization. Furthermore, different cellular responses were observed within aptamer-functionalized regions compared to GelMA regions for at least ten days of culture (Fig. 7), displaying the long-term stability of GFs in these systems. This indicates the aptamers ability to stabilize GFs against various environmental stresses [42] and thereby contributing towards their long-term bioavailability. Similar behavior is observed in nature where upon ECM binding, GFs become stabilized and protected. [43].

Given the simplicity of designing aptamer sequences to capture and release various GFs, this platform is well adaptable for any GF of choice. In complex processes such as angiogenesis, multiple GFs are required at different stages of development. [44]. Specifically, VEGF is responsible for initiating the angiogenesis process and formation of early blood vessels, whereas presence of PDGF-BB ensures their stabilization and maturation. [44]. Owing to aptamers high binding specificities, it is possible to incorporate multiple aptamers within the same hydrogel with different release kinetics for multiple GFs. In outlook, our future studies will focus on achieving mature perfusable vascular networks *in vitro*, by combining both GFs that initiate and stabilize vasculature in a single hydrogel system.

On the longer term, these results, combined with other developments in aptamer technology, provide significant potential for *in vivo* and clinical applications. The platform's ability to induce temporally controlled cell responses by modulating GF bioavailability, provides significant advantages over the currently available strategies for enhancing tissue regeneration within the actively remodeling microenvironment of an implanted engineered tissue. Prior research has revealed the *in vivo* efficacy of intravenous delivery of drugs conjugated with oligonucleotide sequence for targeted refilling of implanted drug depots to mitigate tumors. [45]. Thus, we speculate the possibility to achieve temporally controlled targeted GFs release via intravenous delivery of the external trigger (CS) post-implantation. Alternatively, a local injection of CS could also be used for triggered GF delivery. Notably, the aptamers used in present study were not modified to increase their nuclease resistance. However, various modifications such as PEGylation, locked nucleic acids (LNA), phosphorothioate modification, Spiegelmers (mirror-image L-oligonucleotide aptamers) and g-quadruplex aptamers have been previously reported to enhance their resistance for nucleases-based degradation which is important for long-term *in vivo* application. [46].

Supplementary data related to this article can be found at https://doi.org/10.1016/j.bioactmat.2021.10.024.

The following is the supplementary data related to this article:

Video S2 Video S2

## 4 Conclusion

In conclusion, we demonstrate an aptamer-based dynamic platform for spatiotemporally controlling the bioavailability of an angiogenic GF by exploiting affinity interactions, within a biofabrication friendly polymeric matrix. The platform showcases various outstanding features namely, (i) sustained aptamer incorporation with polymeric matrix via chemical modification, (ii) rapid and localized GF sequestration from culture medium, (iii) triggered GF release via CS hybridization without any non-specific leakage until ten days, (iv) bioactivity for 3D co-cultures, (v) the ability to selectively guide cell responses in 3D, as well as (vi) spatiotemporally regulating tubular morphogenesis of endothelial cells by controlling aptamer-GF bioavailability within bi-phasic hydrogels, similar to native ECM. Moreover, the novel ability to locally manipulate microvascular network formation within bi-phasic hydrogels that are incubated in a single pool of culture medium, opens up new avenues for *in situ* manipulation of cell behavior within aptamer-patterned 3D scaffolds. Altogether, these data provide sufficient proof for harnessing aptamers as biochemical triggers to locally manipulate cell response in 4D, opening up the avenues for creating clinically relevant dynamic biomaterials to be explored for regenerative medicine and tissue engineering applications.

## Author contributions

D.R. and J.R. conceived the project. D.R. designed the study, performed experiments/analysis and prepared the manuscript; A.K. conducted rheological experiments; N.S–N. & I.I. contributed in ELISA data analysis; B.K. and J.R. provided financial and administrative support to the project; J.R. contributed to the designing of experiments and manuscript writing/correction. All authors discussed the results and commented on the manuscript.

## CRediT authorship contribution statement

**Deepti Rana:** Conceptualization, Methodology, Investigation, Formal analysis, Writing – original draft, Writing – review & editing. **Ajoy Kandar:** Formal analysis, Investigation. **Nasim Salehi-Nik:** Formal analysis, Investigation. **Ilyas Inci:** Resources, Formal analysis. **Bart Koopman:** Supervision, Project administration. **Jeroen Rouwkema:** Conceptualization, Writing – review & editing, Supervision, Project administration, Funding acquisition.

## Declaration of competing interest

The authors declare no competing interests.
