# Supplementary material for: Spatiotemporally controlled, aptamers-mediated growth factor release locally manipulates microvasculature formation within engineered tissues
Source: Bioact Mater. 2021 Oct 23;12:71–84. doi: 10.1016/j.bioactmat.2021.10.024 (PMC8777207; doi:10.1016/j.bioactmat.2021.10.024)
Supplement: Multimedia component 1 [file mmc1.docx]

Spatiotemporally controlled, aptamers-mediated growth factor release locally manipulates microvasculature formation within engineered tissues

Deepti Rana^1^, Ajoy Kandar^1^, Nasim Salehi-Nik^1^, Ilyas Inci^2^, Bart Koopman^1^ and Jeroen Rouwkema^1^*

*^1^Department of Biomechanical Engineering, Technical Medical Centre,*

*Faculty of Engineering Technology, University of Twente,*

*7500 AE Enschede, The Netherlands
^2^Izmir Democracy University, Vocational School of Health Services,*

*Department of Dentistry Services, Dental Prosthetics Technology, Izmir, 35140, Turkey*

**Corresponding Author E-mail:* [*j.rouwkema@utwente.nl*](mailto:j.rouwkema@utwente.nl)

**Supporting Information**


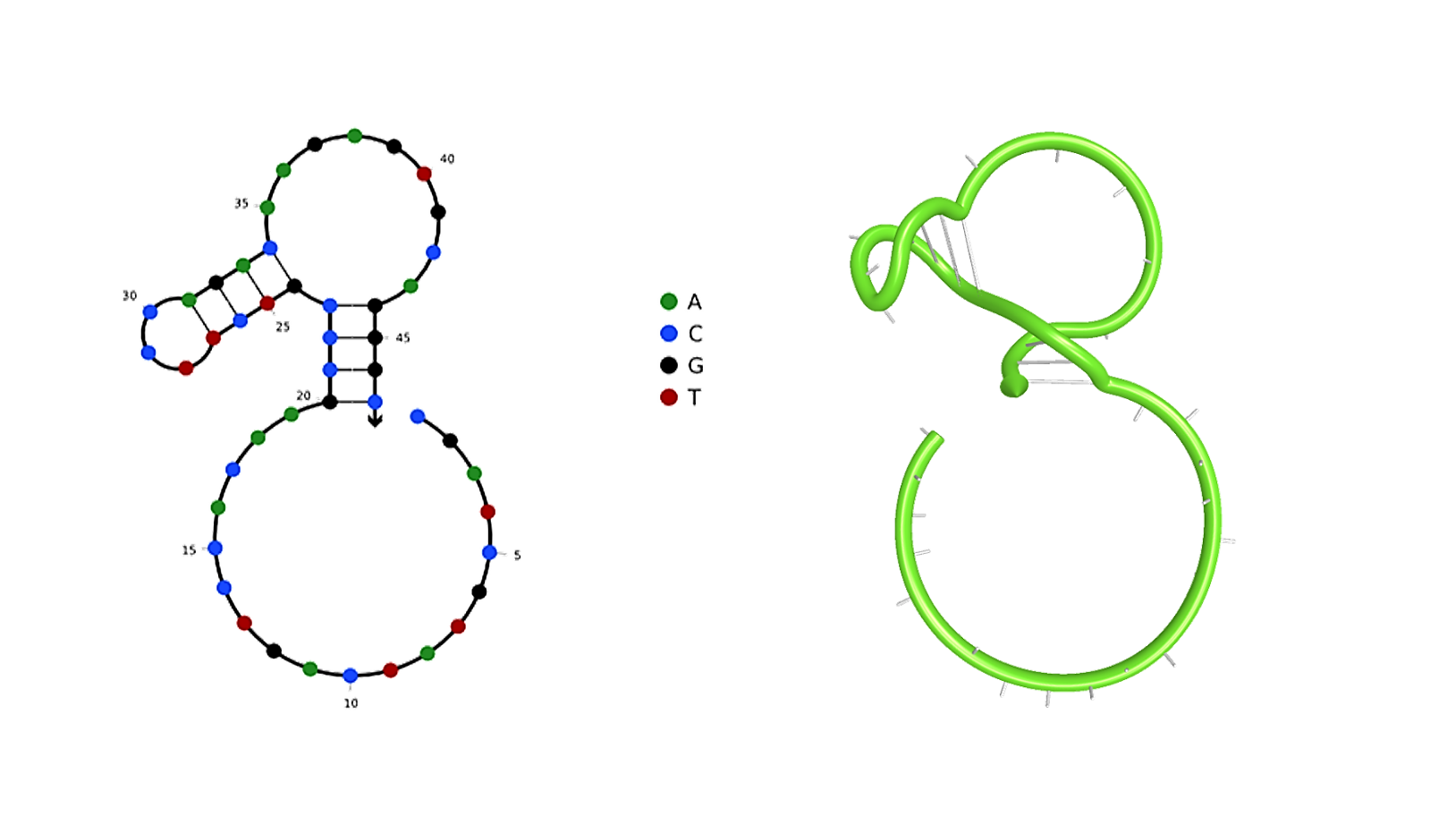


**Figure S1.** Predicted secondary structures and three-dimensional configuration of the VEGF specific aptamer used in this study. The secondary structures of the DNA based aptamers and their three- dimensional conformations were generated using NUPACK software.


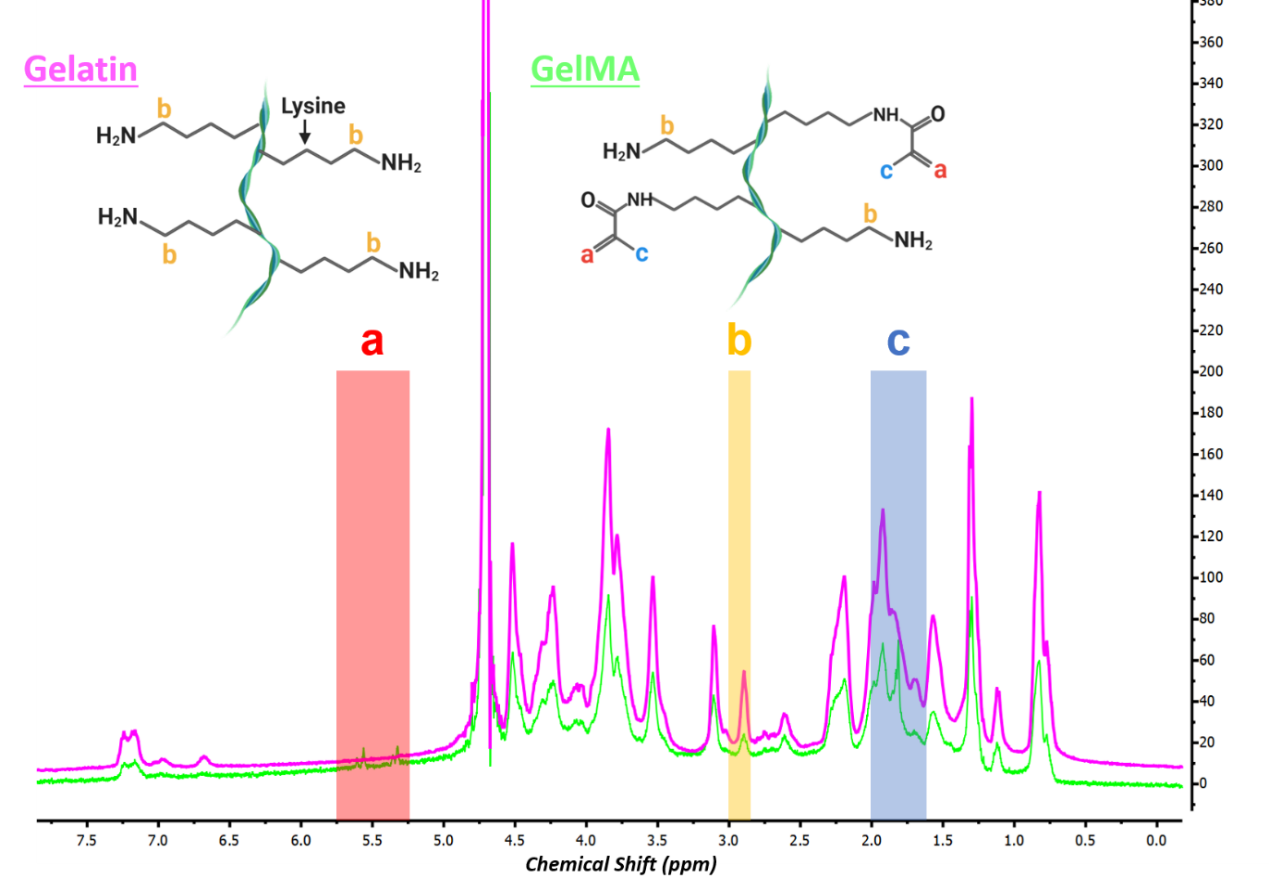


**Figure S2.** NMR Spectrum of gelatin methacryloyl (GelMA) and gelatin. The highlighted red “a” represents the signals of methyl group and yellow “b” shows the acrylic protons of the grafted methacrylic group; and blue “c” indicates the signal of lysine methylene.


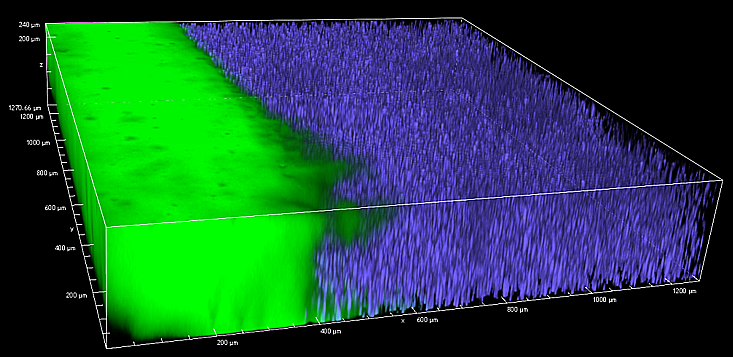


**Figure S3.** 3D projection of acrydite-aptamer functionalized bi-phasic hydrogel after 24hrs incubation with Fluoro-CS at 37ºC. The image is a 3D projection of confocal z-stacks confirming the homogeneous retention of Fluoro-CS throughout the thickness (z = 240µm). To highlight the interface between acrydite-aptamer and GelMA regions, blue fluorescent particles (2µm diameter) were mixed with GelMA pre-polymer solution.


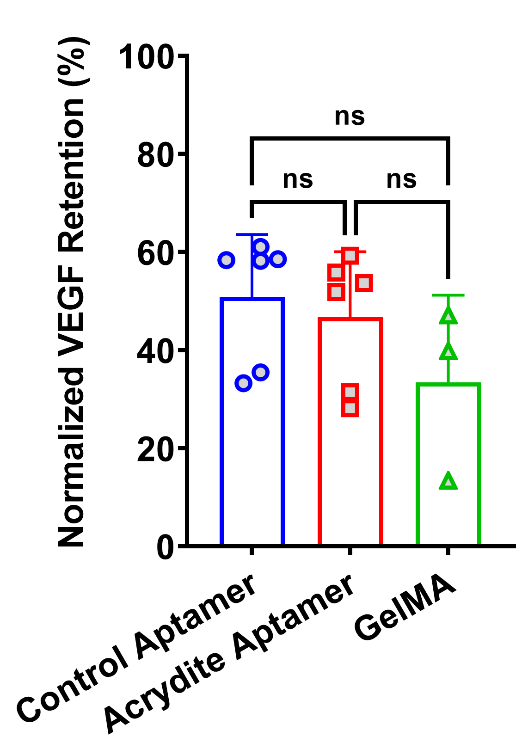


**Figure S4.** The normalized VEGF retention % after 1hr incubation with the control aptamer-, acrydite aptamer-functionalized and GelMA hydrogel samples. The aptamer concentration within hydrogels was fixed at 2.5 nmoles and loaded with 10 ng VEGF in 1ml loading solution. The data is normalized with control PBS samples (data not shown). The quantification was performed using ELISA assay having n=6 (for aptamer samples) and n=3 (for GelMA), experimental replicates. The data is represented as mean ± S.D. with individual data points. The statistical significance was calculated using one-way ANOVA with Tukey’s multiple comparisons test where ns means not significant.


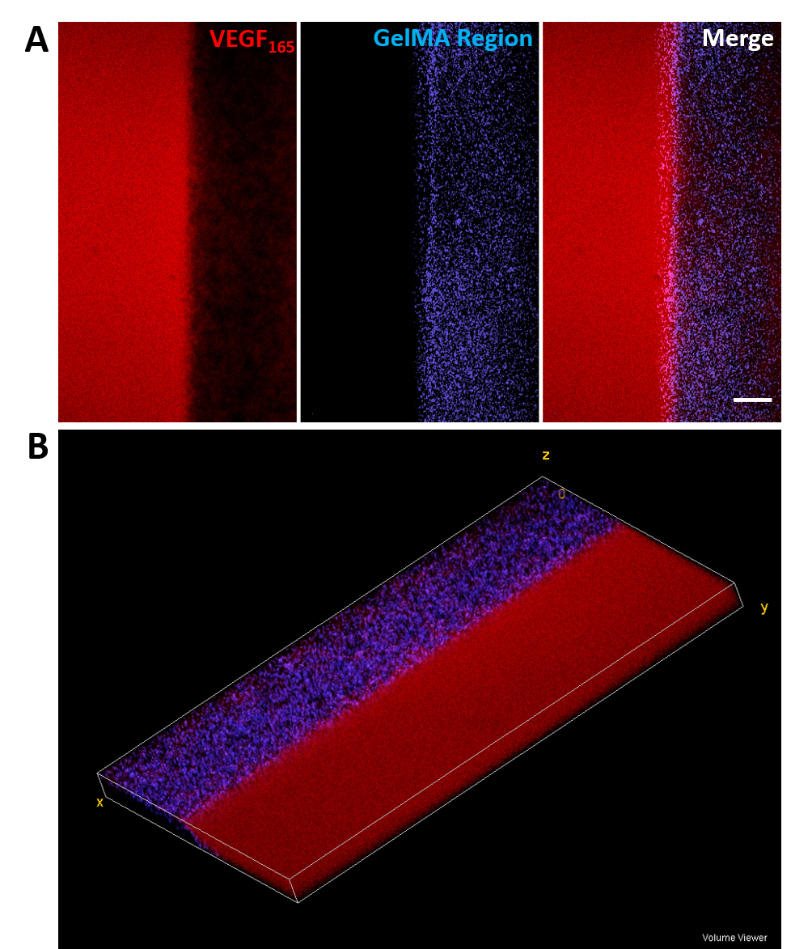


**Figure S5.** Immunostained VEGF_165_ within bi-phasic hydrogels confirming maximum VEGF_165_ sequesteration within aptamer region post 1hr loading. (A) Maximum projection of confocal z-stacks showing VEGF_165_ (red color) within bi-phasic hydrogels having one side with acrydite-aptamer functionalized hydrogels and GelMA region on the other side (blue color). Blue fluorescent beads were mixed with GelMA pre-polymer for identifying the interface. The samples were loaded with VEGF_165_ for 1hr followed by one washing step. Scale bar is 100µm. (B) 3D projection of the confocal z-stack confirming VEGF_165_ presence throughout the thickness (z=160µm).

**
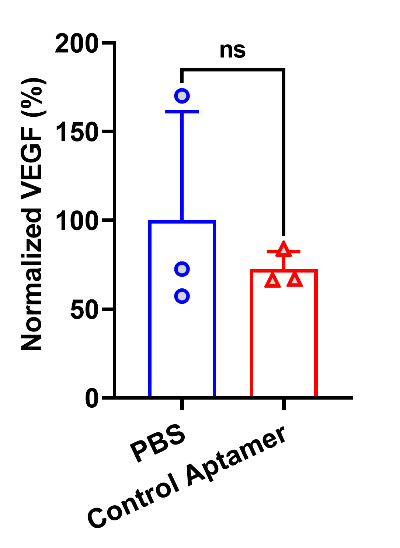
**

**Figure S6.** Normalized VEGF % in presence of control aptamer (2.5 nmoles) and only PBS after 24 hr incubation at 37 °C. The data is normalized with PBS samples. The graph indicates the difference in ELISA sensitivity in VEGF detection in the PBS versus in presence of control aptamers, where (part of) the VEGF will be in a bound state with the aptamer. The quantification was performed using ELISA assay with n=3 experimental replicates. The data is represented as mean ± S.D. with individual data points. The statistical significance was calculated using two-tailed unpaired t-test where ns means not significant.

**
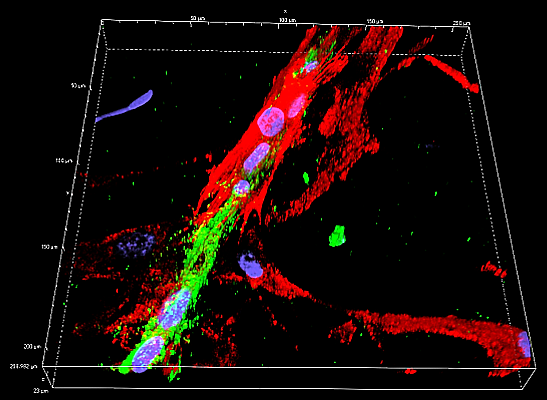
**

**Figure S7.** 3D projection of acrydite-aptamer functionalized bi-phasic hydrogel treated with CS@D4 (Acrydite Aptamer+CS@D4) on day5 at higher magnification (60x objective). Immunostained confocal z-stacks (z = 23µm) expressing von Willebrand factor (vWF) (green) as a marker for endothelial cells and α-smooth muscle actin (α-SMA) (red) as a marker for MSCs differentiation to mural cells, within the HUVECs and MSCs co-cultured samples.


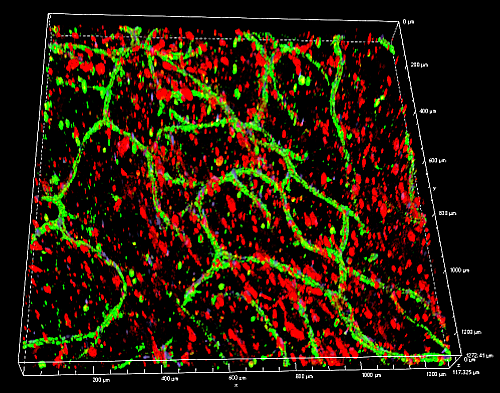


**Figure S8.** 3D projection of acrydite-aptamer functionalized bi-phasic hydrogel treated with CS@D4 (Acrydite Aptamer+CS@D4) on day10 displaying the interface region. Immunostained confocal z-stacks (z = 117µm) expressing von Willebrand factor (vWF) (green) as a marker for endothelial cells and α-smooth muscle actin (α-SMA) (red) as a marker for MSCs differentiation to mural cells, within the HUVECs and MSCs co-cultured samples.


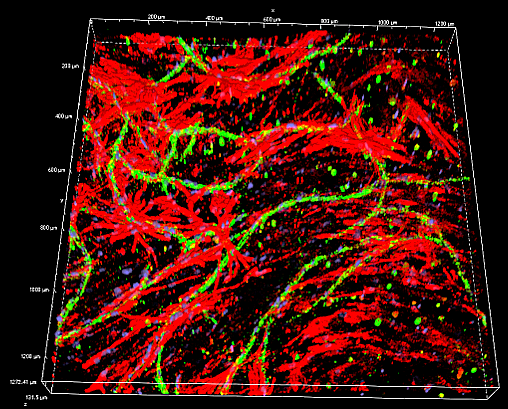


**Figure S9.** 3D projection of control-aptamer functionalized bi-phasic hydrogel treated with CS@D4 (Acrydite Aptamer+CS@D4) on day10 displaying the interface region. Immunostained confocal z-stacks (z = 131µm) expressing von Willebrand factor (vWF) (green) as a marker for endothelial cells and α-smooth muscle actin (α-SMA) (red) as a marker for MSCs differentiation to mural cells, within the HUVECs and MSCs co-cultured samples.


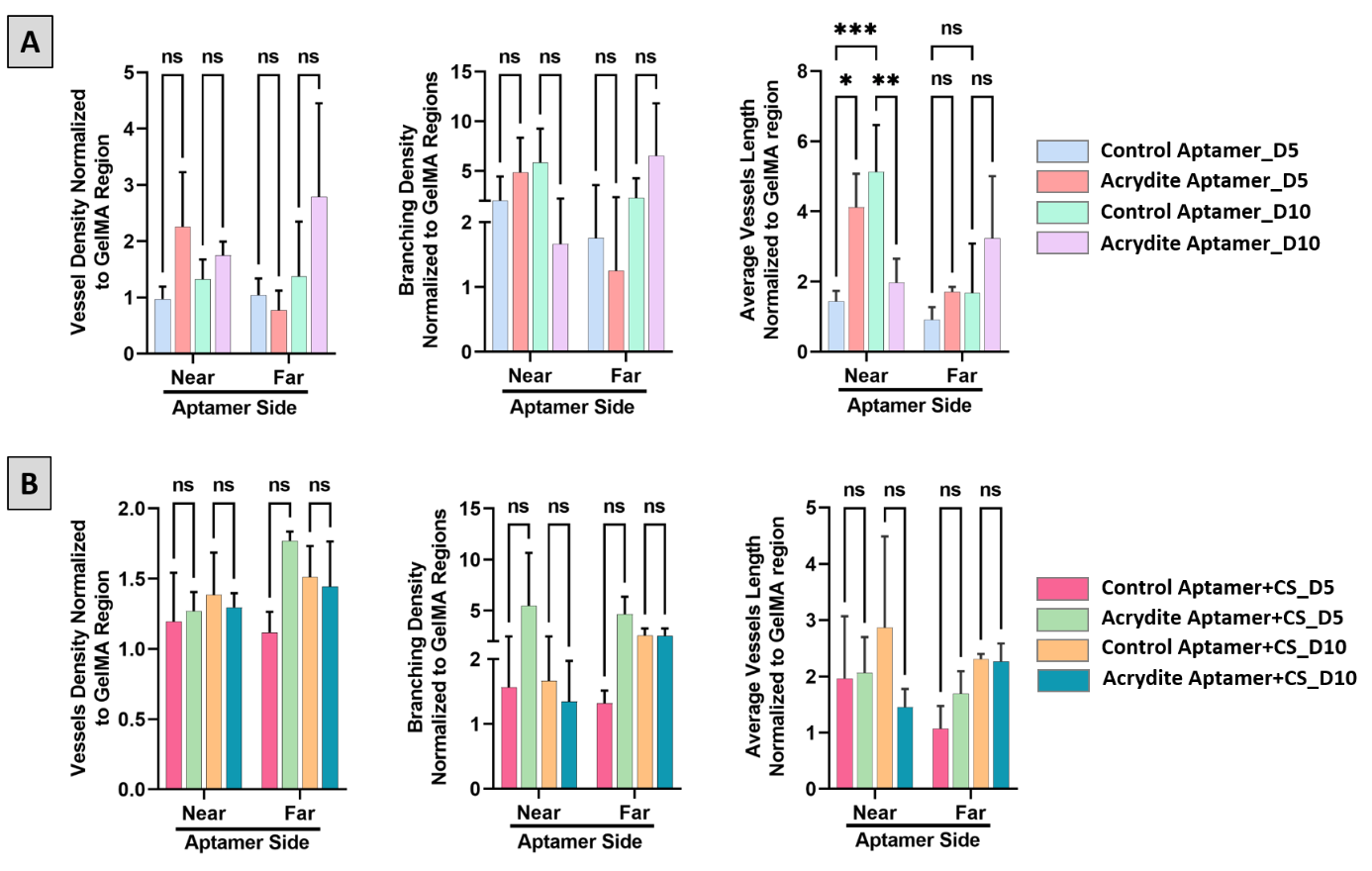


**Figure S10**. Comparative analysis of vWF+ stained vessel network properties within control-aptamer and acrydite-aptamer functionalized bi-phasic hydrogels at different time-points (data as shown in Fig. 7&8). Normalized network properties with their respective GelMA regions where (A) control aptamer samples are compared with acrydite aptamer samples at day5 & day10; and (B) control aptamer samples+CS compared with acrydite aptamer samples+CS on day5 and day10. The values are represented as mean ± SD. The statistical significance was calculated using two-way ANOVA with tukey’s post-hoc test where *p<0.05, **p<0.01, ***p<0.001 and *ns* stands for not significant.

**SUPPORTING TABLES & VIDEO**

**Table S1.** Full sequences and other characteristics of the aptamers used in this study. Tm denotes the melting temperature (50 mM NaCl), MW is molecular weight and N signifies the number of nucleotides.

| Aptamer | Sequence (5’🡪 3’) | Tm | MW | N |
| --- | --- | --- | --- | --- |
| Control Aptamer | CGA TCG TAT CAG TCC ACA AGC CCG TCT TCC AGA CAA GAG TGC AGG GC | 70.8 °C | 14418.4 | 47 |
| Acrydite Aptamer | /5Acryd/CGA TCG TAT CAG TCC ACA AGC CCG TCT TCC AGA CAA GAG TGC AGG GC | 70.8 °C | 14665.6 | 47 |
| Comp. Seq. | CGC CCT GCA CTC TTG TCT GGA AGA CGG GCT TGT GGA CTG ATA CGA TCG | 71.3 °C | 14791.6 | 48 |
| Fluoro – Comp. Seq. | /5Alexa488N/CGC CCT GCA CTC TTG TCT GGA AGA CGG GCT TGT GGA CTG ATA CGA TCG | 71.3 °C | 15487.2 | 48 |

**Video S1.** Confocal z-stack projection of acrydite-aptamer functionalized bi-phasic hydrogel after 24hrs incubation with Fluoro-CS at 37ºC (z = 240µm). To highlight the interface between acrydite-aptamer and GelMA regions, blue fluorescent particles (2µm diameter) were mixed with GelMA pre-polymer solution.

**Video S2.** Immunostained von Willebrand factor (vWF) in green color (endothelial cells marker) and α-smooth muscle actin (α-SMA) in red color indicating MSCs differentiation to mural cells, within the HUVECs and hMSCs co-cultured, acrydite aptamer-functionalized bi-phasic hydrogel showing smooth muscle-like cells wrapping around the developing endothelial network for support.

**Video S3.** Confocal z-stack projection of acrydite-aptamer functionalized bi-phasic hydrogel treated with CS@D4 (Acrydite Aptamer+CS@D4) on day10 displaying the interface region (z = 117µm). Immunostained with von Willebrand factor (vWF) in green color and α-smooth muscle actin (α-SMA) in red color.

**Video S4.** Confocal z-stack projection of control-aptamer functionalized bi-phasic hydrogel treated with CS@D4 (Acrydite Aptamer+CS@D4) on day10 displaying the interface region (z = 131µm). Immunostained with von Willebrand factor (vWF) in green color and α-smooth muscle actin (α-SMA) in red color.
